# Divergence in regulatory mechanisms of GR-RBP genes in different plants under abiotic stress

**DOI:** 10.1038/s41598-024-59341-8

**Published:** 2024-04-16

**Authors:** Yingjie Zhang, Yujian Mo, Junyi Li, Li Liu, Yanhu Gao, Yueqin Zhang, Yongxiang Huang, Lei Ren, Hongbo Zhu, Xingyu Jiang, Yu Ling

**Affiliations:** 1 College of Coastal Agricultural Sciences, Guangdong Ocean University, Zhanjiang, 524088 People’s Republic of China; 2 South China Branch of National Saline-Alkali Tolerant Rice Technology Innovation Center, Zhanjiang, 524088 People’s Republic of China

**Keywords:** GR-RBPa, Abiotic stress, Gene transcription, Alternative splicing, Species-specific, Evolution, Molecular biology, Physiology, Plant sciences

## Abstract

The IVa subfamily of glycine-rich proteins (GRPs) comprises a group of glycine-rich RNA binding proteins referred to as GR-RBPa here. Previous studies have demonstrated functions of GR-RBPa proteins in regulating stress response in plants. However, the mechanisms responsible for the differential regulatory functions of GR-RBPa proteins in different plant species have not been fully elucidated. In this study, we identified and comprehensively studied a total of 34 GR-RBPa proteins from five plant species. Our analysis revealed that GR-RBPa proteins were further classified into two branches, with proteins in branch I being relatively more conserved than those in branch II. When subjected to identical stresses, these genes exhibited intensive and differential expression regulation in different plant species, corresponding to the enrichment of cis-acting regulatory elements involving in environmental and internal signaling in these genes. Unexpectedly, all GR-RBPa genes in branch I underwent intensive alternative splicing (AS) regulation, while almost all genes in branch II were only constitutively spliced, despite having more introns. This study highlights the complex and divergent regulations of a group of conserved RNA binding proteins in different plants when exposed to identical stress conditions. These species-specific regulations may have implications for stress responses and adaptations in different plant species.

## Introduction

Abiotic stresses, including soil salinity, drought, high and low temperatures, have emerged as major limiting factors affecting crop yield and quality^1^. With climate change intensifying and extreme weather events becoming more frequent, it is anticipated that abiotic stresses will increasingly impact crop production, posing a threat to global food security^2,3^. In response to these challenges, plants have evolved a diverse array of molecular mechanisms to rapidly perceive and adapt to environmental changes.

Eukaryotes respond to and adapt to environmental changes by employing transcriptional and post-transcriptional regulatory mechanisms^4^. Alternative splicing (AS) is a process wherein potential splicing sites on precursor mRNA (pre-mRNA) transcripts may or may not be utilized following transcription of the master gene, resulting in the generation of diverse mature mRNA isoforms from an intron-containing gene. AS regulation enhances the diversity of the transcriptome and proteome expressed from the same set of pre-mRNA transcripts^5,6^. Recent data from the human transcriptome and translatome have confirmed that AS can increase protein diversity^7,8^. Similarly, AS contributes to the diversity of the transcriptome and proteome in the plant kingdom^9,10^. Particularly, plants employ more intensive AS regulation to produce protein isoforms adapted to environmental stress^11,12^. Therefore, AS regulation represents an important post-transcriptional strategy in plants' response to environmental stress^13,14^. The application of AS regulation holds promise for enhancing the stress tolerance of crops in the face of global climate change^10,15^.

The majority of post-transcriptional RNA metabolism regulation is mediated by various RNA-binding proteins (RBPs)^16^. GR-RBP proteins represent a subgroup of RBPs within the fourth subfamily of the glycine-rich protein (GRP) superfamily. They are characterized by glycine-rich regions, (Gly)_n_-X (where n is typically an odd number and X can be any amino acid, including glycine), primarily located at the C-terminus of the protein^17,18^. Based on the motifs present in these proteins, GR-RBPs can be further categorized into four subclasses: IVa, IVb, IVc, and IVd. Among these, both IVa and IVd contain RNA recognition motifs (RRMs) at the N-terminus, with the distinction that IVa has one RRM, while IVd has two. Conversely, IVb and IVc have one or more CCHC zinc fingers located at the C-terminus, with IVb featuring an RRM at the N-terminus and IVc featuring a cold shock domain (CSD) at the N-terminus^18,19^. However, the classification of GR-RBP proteins remains unclear and occasionally contentious. For example, AT2G21660 (*AtGRP7*) was initially categorized as a protein in the IVa subgroup^20^, it was recently reclassified as a member of IVb^21^. Similarly, *OsGRP4* and *OsGRP5* in rice, exhibiting strong homology with *AtGRP2/7* in Arabidopsis were initially considered GR-RBP proteins^19,22^. Additionally, GRMZM2G001850 was classified as a GR-RBP protein in maize^23^, though conflicting opinions have been presented regarding the classification of these genes from the two crops^24^.

Previous research has elucidated the critical roles of GR-RBPs, particularly those within the GR-RBPa subfamily, in abiotic stress responses^17,25^. For instance, in Arabidopsis, all seven genes from the AtGR-RBPa subfamily were up-regulated under cold stress among the fifteen GR-RBP genes identified^24–26^. Notably, AT2G21660, one of the AtGR-RBPa genes, was identified as an RNA partner contributing to Arabidopsis' cold tolerance^27^. The heterologous expression of AT2G21660 and AT4G13850 (*AtGRP2*) in rice resulted in an increase in grain yield in transgenic rice^28^. Similarly, Os12g0632000 (*OsGR-RBP4*), one of the six GR-RBPa genes in rice, was induced by salt, heat, and cold treatments^29^. Moreover, Os03g0670700 (*OsGRP3*), another GRP protein in rice with high sequence similarity to *OsGR-RBP4*, positively regulates drought resistance in rice^30^. In tomato, Solyc01g109660.2 (*SlRBP1*) gene has been proposed as a key gene promoting plant growth and development by maintaining normal chloroplast function^31^. Previous studies have also demonstrated that the splicing patterns of AT2G21660 and AT4G39260 in Arabidopsis change according to the circadian rhythm^32,33^. Collectively, these findings suggest that GR-RBPa proteins play roles in plant growth and development, as well as stress responses. However, whether their functions and expression regulation are conserved among different plant species remains unexplored, despite the identification of over 150 GR-RBP genes in plants^34^.

In this study, we selected five plant species: Arabidopsis (*Arabidopsis thaliana*), rice (*Oryza sativa*), maize (*Zea mays*), sweet potato (*Ipomoea batatas*) and tomato (*Solanum lycopersicum*), as materials. These plant species, comprising two monocots and three dicots from four different families, allow for comparisons among species with varying numbers of cotyledons and growth habits. After identifing 34 GR-RBPa proteins from these five the plant species, we constructed a phylogenetic tree to elucidate their evolutionary relationships. Subsequently, we analyzed the functional domains and conserved motifs of these GR-RBPa proteins, along with the chromosomal distribution and characteristics of their encoding genes. After that, we explored and discussed the potential interacting proteins of most of GR-RBPa proteins studied here and their characteristics. We also comprehensively compared the transcriptional regulations and AS patterns of GR-RBPa genes among different plant species under various identical abiotic stresses. In summary, our study characterized GR-RBPa proteins from multiple perspectives and revealed that these crucial RNA-binding proteins undergo species-specific regulations at both transcription and pre-mRNA splicing levels during plant growth and stress responses.

## Materials and methods

### Identification of the GR-RBPa genes

GR-RBPa genes from Arabidopsis, rice, and maize were obtained from the National Center for Biotechnology Information (NCBI) website (https://www.ncbi.nlm.nih.gov/). The GR-RBPa protein from Arabidopsis, encoded by AT2G21660, served as the query sequence. To identify candidate IbGR-RBPa genes in sweet potato, we conducted a BLAST search within the Ipomoea genome hub (https://ipomoea-genome.org/) using the TBtools BLAST program. Candidate SlGR-RBPa genes in tomato were identified by performing a basic local alignment search tool (BLAST) search with default parameters on the Solanaceae Genomics Network website (https://solgenomics.net/). Finally, we defined target GR-RBPa genes in sweet potato and tomato based on the typical structural characteristics of the GR-RBPa gene subfamily using online tools such as SMART (http://smart.embl.de/) and the NCBI Conserved Domain Database (CDD, https://www.ncbi.nlm.nih.gov/cdd).

### Analyses of phylogeny, protein properties and multiple sequence alignment

The sequences of the target GR-RBPa proteins from the five plant species were compared using ClusterW tool in MEGA 11.0 software. Subsequently, a phylogenetic tree was constructed using the Neighbor Joining method with 1000 bootstrap replications and other default parameters implemented in MEGA 11.0^35^.

The physical and chemical properties (Supplemental Table 1), including amino acid number, molecular weight (MW), theoretical isoelectric point (pI), and hydrophobic value, of the five plant GR-RBPa proteins were determined using the online tool ExPASy (http://expasy.org/tools/)^36^. Sequence alignment of these proteins was performed using DNAMAN software with default parameters.

### Analysis of gene structure, protein domain and conserved motif

The gene structure was conducted using the online tool GSDS (http://gsds.cbi.pku.edu.cn/index.php), based on previous report^37^. Protein structures were predicted using SMART and NCBI-CDD. The protein domains of GR-RBPa were visualized using TBtools. Conservative motifs of each protein were further analyzed through online tools MEME (https://meme-suite.org/meme), with the predicted number of conservative motifs as 9 and other parameters set as default.

### Prediction of protein interaction networks

The protein–protein interaction networks of GR-RBPa proteins from different species were analyzed using the online program STRING (https://string-db.org) with default parameters^38^.

### Secondary structure analysis of protein

To predict protein products from different alternatively spliced mRNA isoforms, the largest open reading frame (ORF) of each transcript was utilized as the potential coding region. The secondary structure of the resulting amino acid sequence was generated using the online software SWISS-MODEL (https://swissmodel.expasy.org/).

### Chromosome localization, cis-elements and syntenic analysis

The location of GR-RBPa genes were determined based on annotation information obtained from five plant genome networks. The General Feature Format version 3 (GFF3) file was used to extract the sequence coordinates of all mRNA introns within the genome sequences of the five plants by identifying exon positions to infer intron positions. Prediction of cis-regulatory elements in the promoter region (2 kb upstream of the gene) and introns of each GR-RBPa gene was carried out using the online software PlantCARE (http://bioinformatics.psb.ugent.be/webtools/plantcare/html/)^39,40^. Heat shock response elements (HSEs) were manually searched for by referencing an earlier report^41^. Screening and visualization of the results were conducted using TBtools.

Syntenic analysis and visualization of GR-RBPa genes among the five plants were performed using MCScanX and the Multiple Synteny Plot, respectively, within the TBtools software.

### Plant materials and stress management

Seedlings of Arabidopsis (*Arabidopsis thaliana*, Col-0), rice (*Oryza sativa*, IR29), maize (*Zea mays*, Huanuo 2), sweet potato (*Ipomoea batatas*, Jishu 26), and tomato (*Solanum lycopersicum*, Nong-Bo-Fen 3) were previously hydroponically cultured in a growth room at 25 °C under a 12/12 light/dark cycle. Following this, the seedlings were transferred to medium solutions containing 200 mM NaCl for salt and 20% PEG-6000 for drought stress treatment. For temperature stress, the seedlings were exposed to growth chambers with temperatures set at 42 °C for heat stress and 4 °C for cold stress. The durations of these stress treatments were 3 h, 6 h, and 24 h, respectively. Leaf samples were collected and immediately frozen with liquid nitrogen for the following RNA extraction. For phenotypic analysis, the seedlings were returned to a normal medium solution and cultured under appropriate growth conditions of 25 °C and a 12/12 light/dark cycle.

### RNA extraction, RT-qPCR and semi-qPCR

Total RNA was extracted using an RNA extraction kit (Cat#9769, Takara) following the manufacturer's instructions. Subsequently, cDNA synthesis was carried out with a cDNA synthesis kit (R323, Vazyme, Nanjing, China). PCR and qRT-PCR primers were designed using SnapGene software (the primer sequences are provided in Supplemental Tables 2 and 3, respectively). For RT-PCR, the reaction volume was 20 µL, consisting of 10 µL of 2 × Hief® Robust PCR Master Mix (Cat#101016ES03, YESEAN, Shanghai, China), 1 µL of primer-F (10 µM), 1 µL of primer-R (10 µM), 1 µL of cDNA, and 7 µL of distilled water. The RT-PCR program was as follows: 3 min at 95 °C, 40 cycles of 30 s at 95 °C, 30 s at 58 °C, and 2 min at 72 °C, with a final extension at 72 °C for 5 min. For qRT-PCR, a 2 × Hief® qPCR SYBR® Green Master Mix (Cat#11201ES08) was obtained from YESEAN (Shanghai, China). The 20 μL reaction volume contained 10 μL of 2 × Hief® q-PCR SYBR® Green Master Mix, 0.2 µL of primer-F (10 µM), 0.2 µL of primer-R (10 µM), 0.5 µL of cDNA, and 4.1 µL of distilled water. The reaction was carried out in the CFX Connect™ Real-Time System with the following procedure: 30 s at 95 °C, 40 cycles of 10 s at 95 °C, and 30 s at 60 °C. Internal reference genes were used according to previous reports^42–46^. The primer sequences for these reference genes are listed in Supplemental Table 4. Gene expression levels were calculated using the 2^(− ΔΔCt) method^47^. Statistical analysis was performed using a Student's t-test, and differences were considered significant when P < 0.05.

### Statement of plant collection and use

The plant collection and use was in accordance with all the relevant guidelines.

## Result

### Identification of GR-RBPa genes from monocot and dicot species

The sequences of GR-RBPa genes from Arabidopsis, rice, and maize (7, 6, and 6) were directly obtained from NCBI^24^. To identify candidate GR-RBPa genes in sweet potato, a sequence search was performed using the protein sequence of Arabidopsis AT2G21660 as a query in the Ipomoea genome hub. Subsequently, the resulting candidates were screened using the SMART and NCBI-CDD online tools to confirm the presence of an RNA recognition motif (RRM) domain and glycine-rich regions, which are fundamental features of GR-RBPa proteins.

As a result, seven genes were identified as GR-RBPa genes in sweet potato (labeled as IbGR-RBPas) with the database IDs g34073, g43136, g14599, g51434, g30047, g53259, and g7929, respectively. A similar approach was employed to identify eight SlGR-RBPa genes from the Solanaceae Genomics Network, with database IDs Solyc10g051390.1, Solyc10g051380.1, Solyc02g088790.2, Solyc02g066930.2, Solyc10g081180.1, Solyc01g109660.2, Solyc05g053780.2, and Solyc09g092320.2.

In summary, the number of GR-RBPa genes identified in the five plant species remained relatively consistent, ranging from 6 to 8. This suggests that the proliferation and loss of GR-RBPa family members are relatively conserved in different plant species.

### Phylogeny and physicochemical properties of GR-RBPa proteins

**Figure 1:**
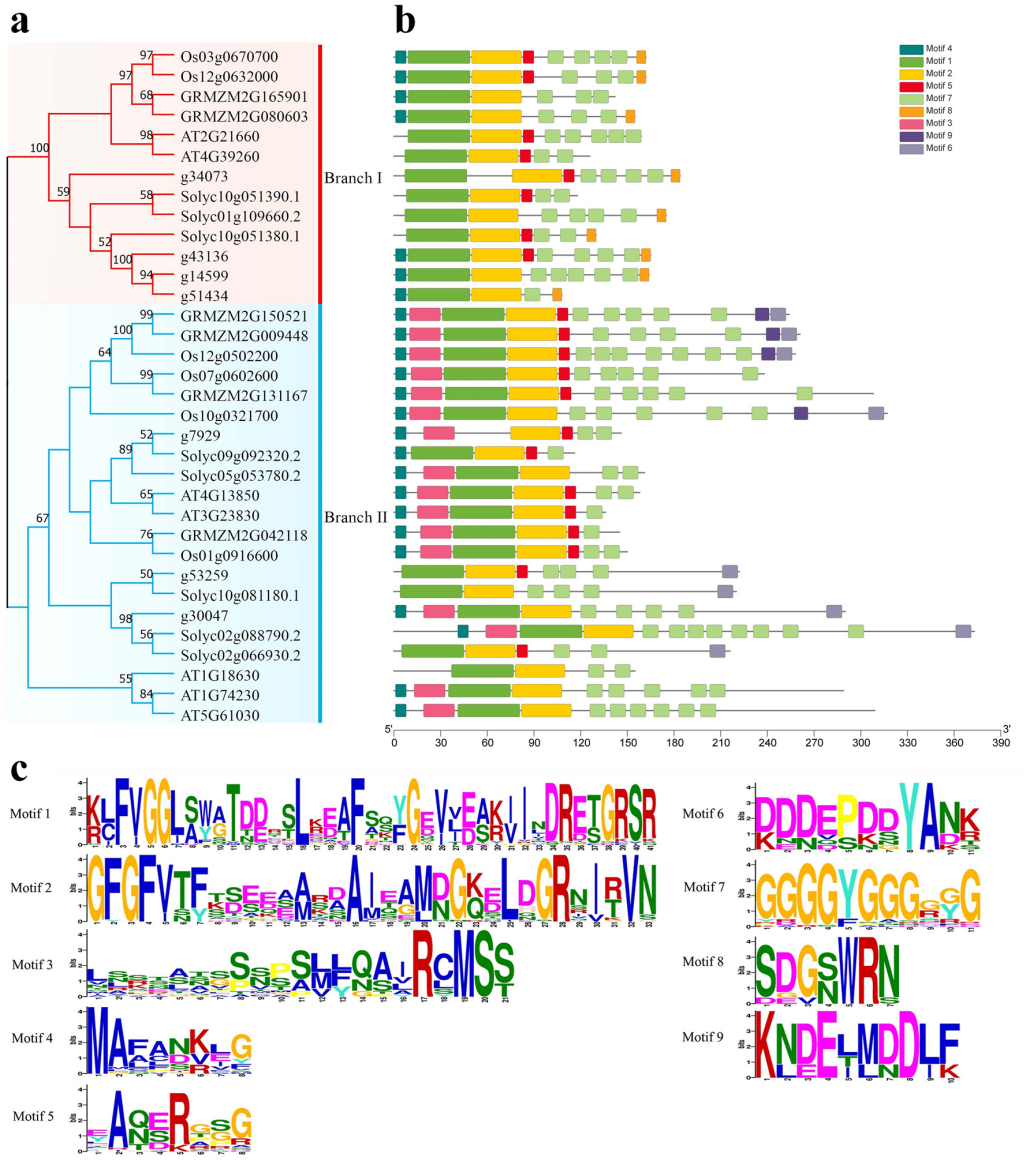
Phylogenetic tree, protein structure, and conserved protein motifs of GR-RBPa genes across five plant species. (**a**) Phylogenetic tree depicting GR-RBPa proteins extracted from five plants, with branch I highlighted in red and branch II in blue. (**b**) Composition of conserved motifs in GR-RBPa proteins across the five plant species. Conserved sequences numbered 1–9 are depicted by differently colored boxes. (**c**) Analysis of the nine conserved motifs, with the vertical axis representing the conservation rate and the horizontal axis indicating the conserved sequence domain of each amino acid. Higher frequency is indicated by larger letters.

A total of 34 GR-RBPa protein sequences from the five plant species were aligned using MEGA11.0 to create a phylogenetic tree. Figure 1a displays the gene IDs. Based on the phylogenetic relationships of these plants, the GR-RBPa subclass proteins are divided into two major branches: branch I and branch II (Fig. 1a). The following proteins were grouped into branch I, indicating high homology among these 13 members, which are Os03g0670700, Os12g0632000 from rice, GRMZM2G165901 and GRMZM2G080603 from maize, AT2G21660 and AT4G39260 from Arabidopsis, g43136, g14599, g51434 and g34073 from sweet potato, and Solyc10g051390.1, Solyc10g051380.1 and Solyc01g109660.2. The remaining 21 members were sub-grouped into branch II.

The physicochemical properties of the GR-RBPa proteins are presented in Supplemental Table 1. Among the 13 GR-RBPa members in branch I, the number of amino acid (AA) ranged from 108 AA (g51434) to 184 AA (g34073), with molecular weights ranging from 11.88 kDa (g51434) to 19.00 kDa (g34073). In branch II, some members have significantly more amino acid residues. The length of the 21 GR-RBPa members in branch II ranged from 116 AA (Solyc09g092320.2) to 373 AA (Solyc02g088790.2), with molecular weights between 12.22 kDa (Solyc09g092320.2) and 38.30 kDa (Solyc02g088790.2). Furthermore, only one GR-RBPa protein (Solyc10g051380.1) in branch I is alkaline, while six proteins in branch II are alkaline. These alkaline proteins are from sources including Arabidopsis, rice, sweet potato, and tomato. It is noteworthy that all 34 GR-RBPa proteins have negative GRAVY values, indicating that they are hydrophilic proteins.

### Conservative motif and functional domains of GR-RBPa proteins

Analysis of conserved motifs in GR-RBPa proteins in plants provides valuable insights into their potential functions.

In this study, the conserved motifs of the 34 GR-RBPa proteins were analyzed using the online software MEME, leading to the identification of a total of 9 conserved motifs (Fig. 1b,c). Notably, all 34 members contained 3 highly conserved motifs: a 41-amino acid motif (motif 1), a 33-amino acid motif (motif 2) and an 11-amino acid motif (the glycine-rich motif 7) (Supplemental Table 5). The presence of these highly conserved motifs suggests that these GR-RBPa proteins may share similar molecular functions. It is confirmed that motif 1 and motif 2 constitute the N-terminal RNA recognition motif (RRM) domain of GR-RBPa protein (Supplemental Fig. 1). Importantly, motif 8 was found exclusively in 9 proteins belonging to branch I (Os03g0670700, Os12g0632000, GRMZM2G080603, g30473, Solyc01g109660.2, Solyc10g051380.1, g43136, g14599, g51434), while motif 3, motif 6, and motif 9 were specific to GR-RBPa proteins in branch II. This observation suggests that there may be functional variations within the GR-RBPa gene subfamily across different branches.

**Figure 2:**
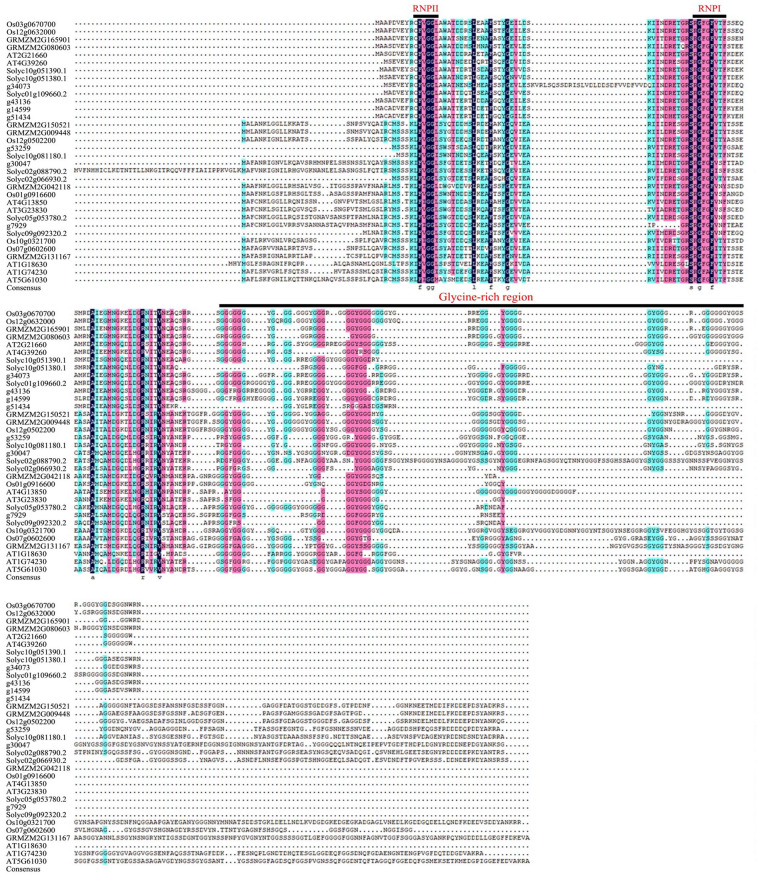
Multiple sequence alignment of the GR RBPa proteins across five plant species. The N-terminus features two highly conserved regions, denoted by RNP-2 and RNP-1 indicated by a solid line above their sequence. The C-terminus includes a glycine-rich region highlighted by a solid line.

Multiple sequence alignment through DNAMAN demonstrated the conservation and variation of each GR-RBP protein at the single amino acid resolution, also confirming the presence of a relatively conserved RRM domain at the N-terminus and a less conserved glycine-enriched C-terminus (Fig. 2).

### Analysis of protein partners of GR-RBPa proteins

**Figure 3:**
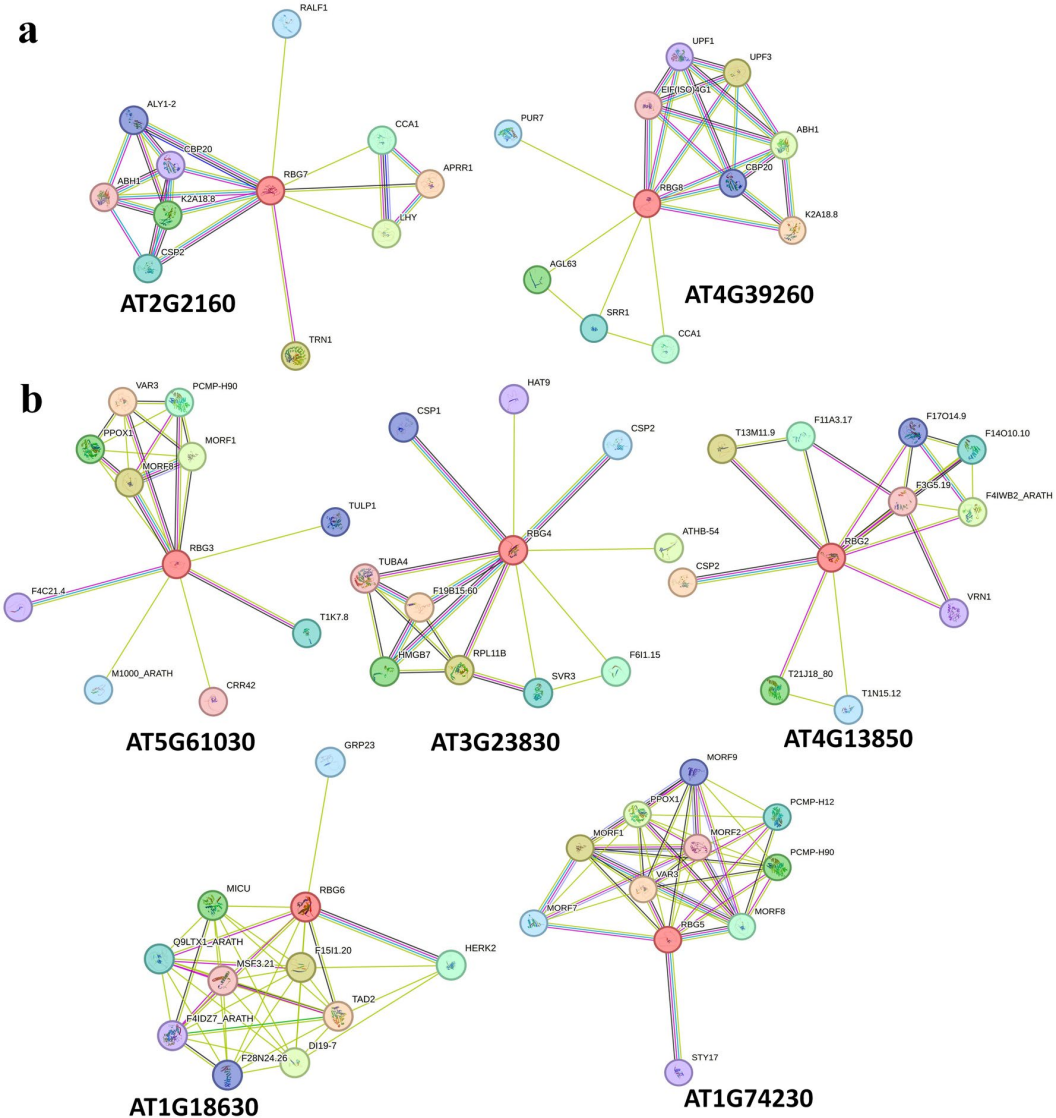
Protein–protein interaction diagram of GR-RBPa genes in Arabidopsis. (**a**) Protein interaction network of branch I GR-RBPa proteins in Arabidopsis. (**b**) Protein interaction network of branch II GR-RBPa proteins in Arabidopsis. Nodes in different colors represent query proteins and their first shell of interactors. Filled nodes indicate that the 3D structure is known or predicted. Colored lines between nodes represent known or predicted interactions between the respective proteins.

Potential interacting targets of GR-RBPa proteins were searched in the STRING database. Our results demonstrated that a substantial number of GR-RBPa-interacting proteins were those involved in RNA processing, particularly in pre-mRNA splicing control (Fig. 3, Supplemental Fig. 2). For example, two pre-mRNA splicing regulators, ABH1 and CBP20, were found in Arabidopsis and rice, splicing factors Q6AVF4 and Q6ETX3 were found in rice, A0A1D6QP33, A0A1D6Q0L8, SC26 were found in maize, and A0A3Q7EGR2, Q53U41 were found in tomato. Additionally, it was found that GR-RBPa proteins are prone to interact with cold shock proteins (CSPs) in Arabidopsis. Potential proteins encoded from reported cDNA clone sequences, such as Q8LHL0, Q6ZDR7, Q6ZGM1, Q6YX88, and so on, were considered to interact with GR-RBPa proteins in rice. GR-RBPa proteins in maize also interact with stress-responsive proteins, such as disease-resistant proteins A0A1D6J341, B6STS5, peroxidase Gpm853, Uaz235 (px), and so on. A large number of RRM domain-containing proteins were considered to interact with GR-RBPa proteins in tomato, suggesting interaction between RNA binding proteins. It is worth noting that, except for rice, in the same plant species, the GR-RBPa proteins in branch I share more interacting proteins with each other compared to the situation in branch II (Fig. 3a, Supplemental Fig. 2).

This finding indicates that GR-RBPa proteins could have a broad array of interacting partners involving in regulating plant growth and stress response. Many of these partners are associated with RNA processing and pre-mRNA splicing control, indicating that GR-RBPa proteins may play a role in regulating gene expression at the post-transcriptional level, particularly in mRNA splicing and processing. Thus, the interacting proteins between different GR-RBPa proteins changed obviously, especially in branch II.

### Structure analysis of GR-RBPa genes

**Figure 4:**
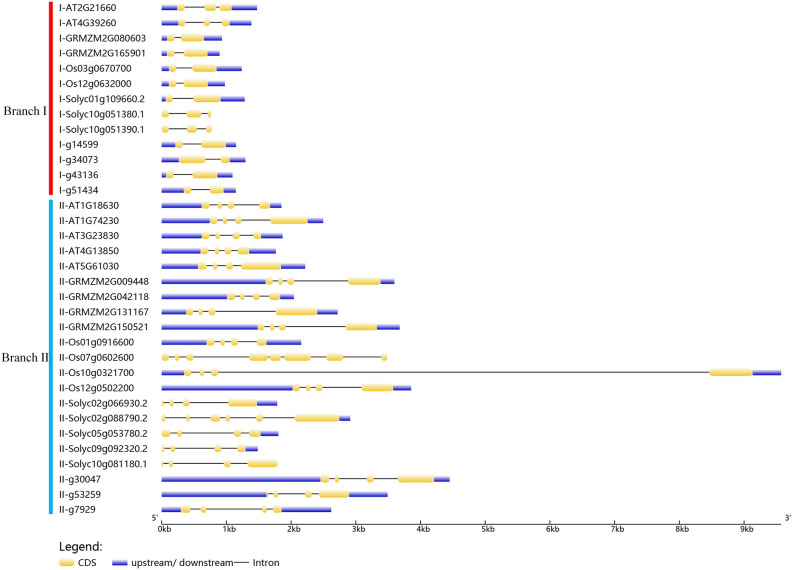
Gene structure of GR-RBPa genes in the five plant species. The blue box denotes the untranslated 5ʹ and 3ʹ regions, the yellow box represents exons, and the black line indicates introns.

Gene structure analysis was performed using the online program GSDS (Fig. 4). The analysis revealed that most of the GR-RBPa genes in branch II exhibited longer gene structures compared to those in branch I. Specifically, 76.92% of branch I members had only one intron, while two genes from Arabidopsis (AT2G21660 and AT4G39620) and two genes from tomato (Solyc10g051390.1 and Solyc10g051380.1) contained two introns each. In contrast, the majority of branch II members (90.48%) had three introns, with exceptions such as Solyc02g088790.2 and Os10g0321700, which contained five and seven introns, respectively. It's worth noting that, a subset of genes, primarily from tomato, lacked 5ʹ UTR or both 5ʹ- and 3ʹ UTRs, as indicated by the analysis of sequences obtained from the genome sequence databases of these plants.

### Cis-regulatory elements of GR-RBPa genes

**Figure 5:**
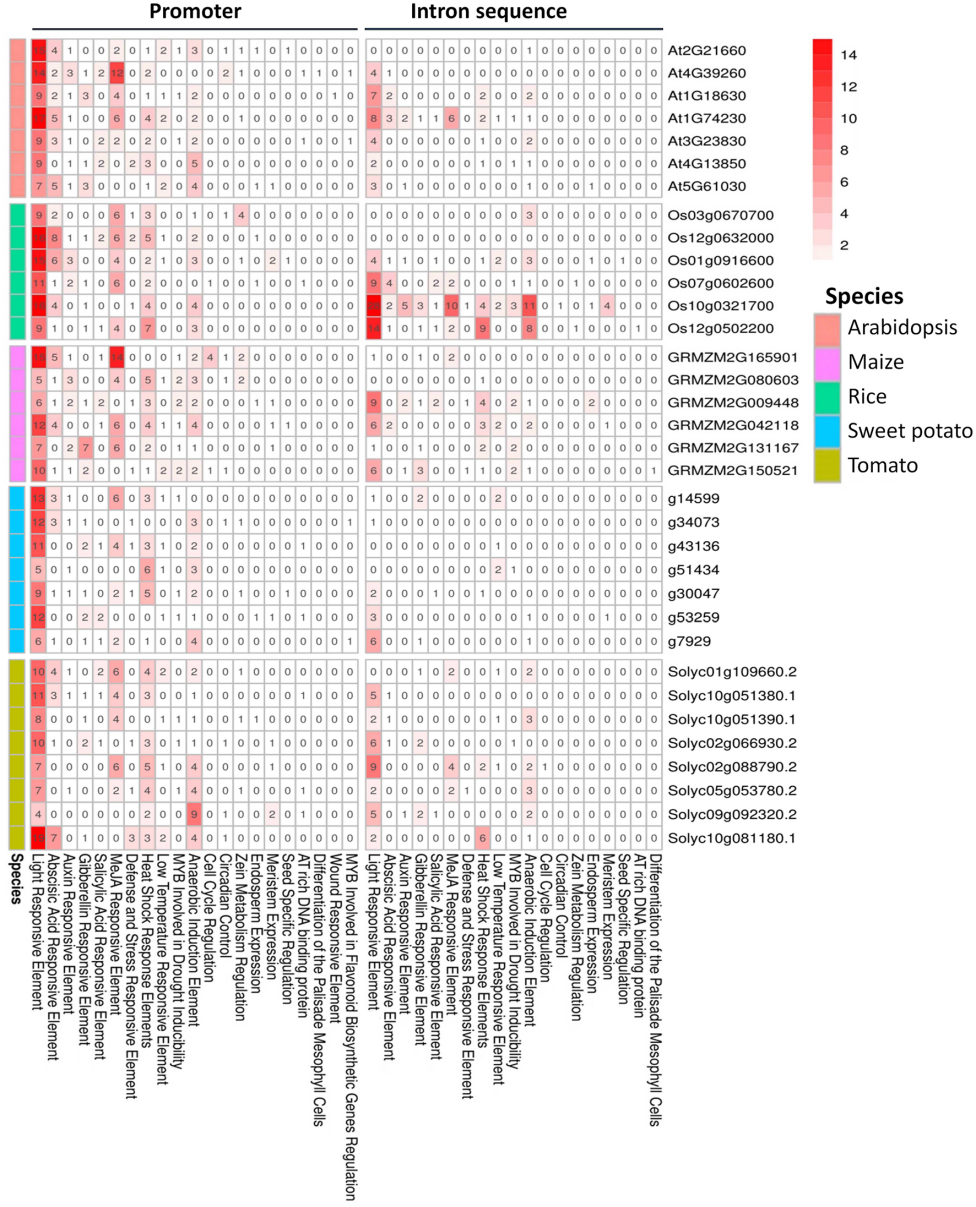
Analysis of cis-acting elements in promoters and introns of GR-RBPa subclass genes from the five plant species. The number of cis-regulatory elements in AtGR-RBPa, OsGR-RBPa, ZmGR-RBPa, IbGR-RBPa, and SlGR-RBPa genes is depicted. Increasing intensities of red color correspond to increasing numbers of cis-acting elements. White indicates the absence of cis-regulatory elements. Arabic numerals denote the number of elements contained.

Analyzing cis-regulatory element is crucial for understanding gene expression regulation. In this study, we investigated cis-elements in promoters (2 Kb sequences upstream of the GR-RBPa gene) and introns of GR-RBPa gene. These sequences were then analyzed using the PlantCARE online software (Fig. 5).

Our findings revealed that light response elements were prominently accumulated in the promoters and intronic sequences of most GR-RBP genes, indicating that the expression of GR-RBPa genes might be controlled by light. Additionally, cis-regulatory elements associated with various hormones such as auxin, abscisic acid, gibberellin, and salicylic acid were present in the promoters of most GR-RBPa genes, suggesting their potential involvement in hormone-related responses. Furthermore, cis-elements related to different stresses, including responses to high and low temperatures as well as anaerobic conditions, were commonly found in the promoters of GR-RBPa genes. A limited number of cis-regulatory elements associated with endosperm expression, meristem regulation, seed-specific control, circadian rhythm, and cell cycle regulation were identified in both promoters and introns. Intronic sequences of GR-RBPa genes possess lower numbers of cis-elements than their promoters in general. However, introns of Os10g0321700 and Os12g0502200 contained more light and anaerobic responsive elements than their promoters, respectively. Also, introns of Os12g0502200 and Solyc10g081180.1 possessed more heat responsive elements than their promoters, respectively. The abundance of cis-regulatory elements in promoters and intronic sequences of GR-RBPa genes indicates that these genes would participate in response to various stresses.

### Syntonic analysis of GR-RBPa genes

The GR-RBPa genes are distributed on chromosomes with varied sizes in each plant species. Analysis revealed that AtGR-RBPa genes are present on all five chromosomes of *Arabidopsis thaliana*. In sweet potato and tomato, GR-RBPa genes are distributed on chromosomes 2, 4, 7, 9, 11, and 13, and chromosomes 1, 2, 5, 9, and 10, respectively. In rice and maize, the two monocots, OsGR-RBPa genes are located on chromosomes 1, 3, 7, 10, and 12, while ZmGR-RBPa genes are found on chromosomes 1, 3, 5, 7, 8, and chromosome 10 (Supplemental Table 1).

**Figure 6:**
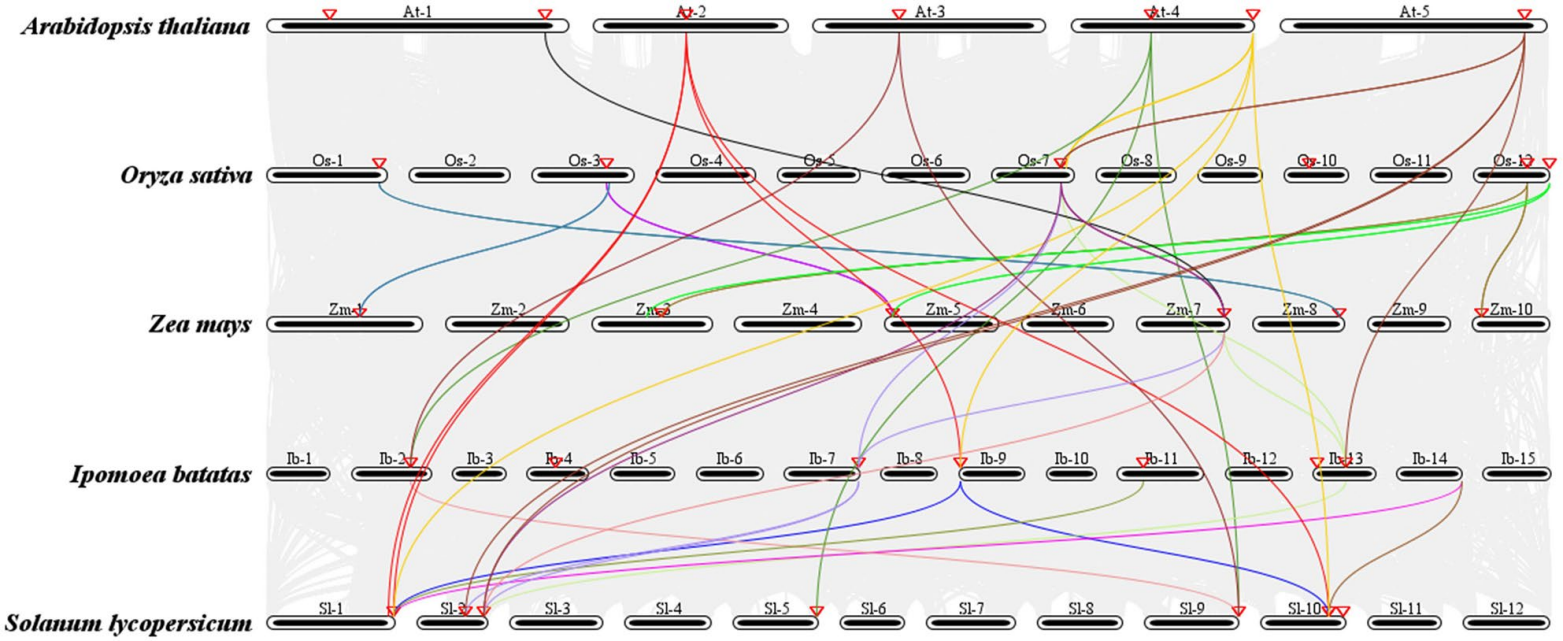
Synteny analysis of GR-RBPa subfamily genes among the five plant species. Gray lines in the background represent collinear blocks within the Arabidopsis, rice, maize, sweet potato, and tomato genomes, while colored lines highlight syntenic GR-RBPa gene pairs. The red triangle denotes the position of the GR-RBPa gene on the chromosome.

To further understand the evolutionary relationships of GR-RBPa genes among the five plant species, a co-lineage analysis was conducted (Fig. 6, Supplemental Table 6). The numbers of orthologous gene pairs varies, with 10, 5, 9, and 8 pairs observed in comparisons between Arabidopsis and tomato, Arabidopsis and sweet potato, tomato and sweet potato, and rice and maize, respectively. However, comparisons between dicots and monocots have significantly fewer orthologous gene pairs, with only 1 or 2 pairs identified in each dicot-monocot comparisons. This indicates a clear evolutionary distinction between GR-RBPa genes in dicots and monocots.

### Differential phenotypic responses of plants to the same stresses

Previous reports have demonstrated that GR-RBPa proteins participate in various physiological responses, including salt, cold, high temperature, and drought in different plants species^17,25^. However, the response of homologous GR-RBPa genes from diverse plant species to identical stresses has not yet been studied before. To address this gap, we subjected the five plant species to the same salt, drought, heat, and cold stress treatments in a water-based culture.

**Figure 7:**
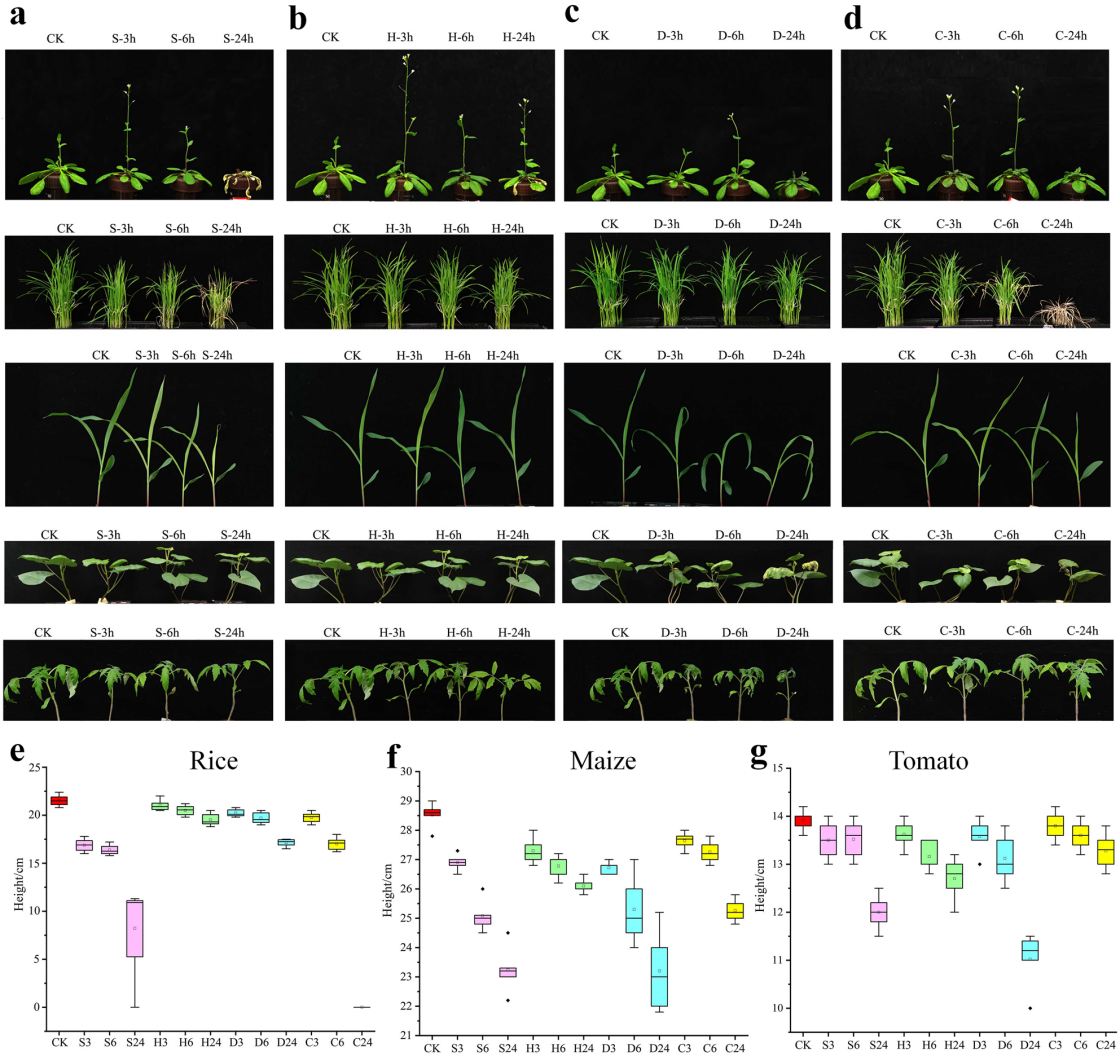
Phenotypic analysis of the five plant species after exposure to abiotic stresses. (**a**) Salt stress. (**b**) Heat stress. (**c**) Drought stress. (**d**) Cold stress. The labels 3 h, 6 h, and 24 h represent 3 h, 6 h, and 24 h, respectively. Panels (**e**–**g**) show variations in the heights of rice, maize, and tomato, respectively, after different treatments.

In our experiment, all plants initially exhibited healthy growth with no discernible phenotypic defects in the original culture solution. However, as the duration of salt, drought, heat, and cold treatments increased, the stress-induced inhibitions on plant growth became more evident (Fig. 7). Salt stress treatments significantly inhibited plant growth, particularly in rice and Arabidopsis (Fig. 7a,e). Exposure to 42 °C did not cause clear damage to plant growth and development; however, a slower growth rate was observed in the group subjected to 24 h of heat stress (Fig. 7b). Simulated drought stress resulted in severe damage in maize, sweet potato, and tomato, with leaves noticeably wilting after only 3 h of treatment. In contrast, the damage caused by drought stress in Arabidopsis and rice was less severe compared to the other plants (Fig. 7c). Arabidopsis and tomato exhibited greater tolerance to cold stress in our experiment, showing less damage and growth inhibition, while rice was found to be the most sensitive among the five plant species, with complete death of rice seedlings observed after 24 h of exposure (Fig. 7d,e,g).

### Differentiated transcriptional regulations of GR-RBPa genes

**Figure 8:**
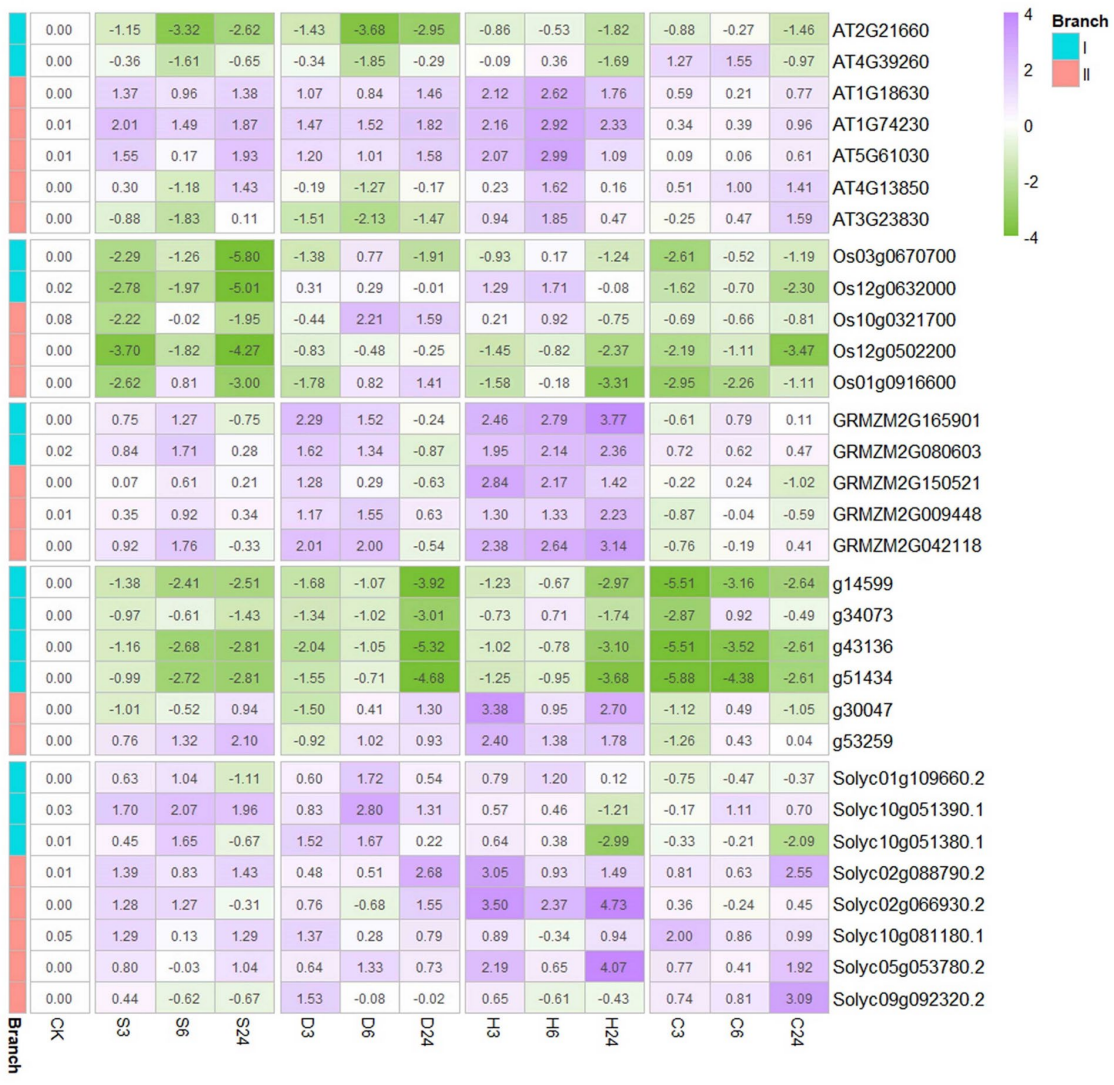
Expression Changes of GR-RBPa Genes in Response to Abiotic Stresses. The relative expression levels of GR-RBPa genes are presented in 2^(x) style, where the expression level of each gene under normal temperature conditions is set to 1, represented as 2^ (0). The number and corresponding color in each cell in the heatmap indicate the intensity of induction (purple) or suppression (green) of gene expression compared to its expression level under normal temperature conditions. CK represents 0 h of stress treatment. S denotes salt stress, D represents drought stress, H indicates heat stress, and C denotes cold stress. The numbers "3", "6", and "24" represent the treatment duration of 3 h, 6 h, and 24 h, respectively.

We then examined the expression patterns of GR-RBPa genes from different plant species in response to various environmental stresses. We detected all 13 genes from branch I and a total of 18 members from branch II. As shown in Fig. 8 and Supplemental Table 7, noticeable changes in the expression patterns of GR-RBPa genes were observed among different plant species when subjected to the same abiotic stresses.

In Arabidopsis, GR-RBPa genes in branch I exhibited a tendency to be down-regulated by abiotic stresses, especially osmotic stresses like salt and drought, with the exception of AT4G39260, which showed up-regulation in response to short periods of cold stress (3 h and 6 h). In branch II, AT1G18630, AT1G74230, and AT5G61030 were up-regulated in response to various stresses, whereas AT4G13850 and AT3G23830 were significantly down-regulated by osmotic stresses but generally up-regulated by temperature stresses. In rice, GR-RBPa genes tended to be down-regulated by salt and cold stresses, while exhibiting inconsistent regulation in response to drought and heat. In maize, all tested GR-RBPa genes tended to be up-regulated by short-term salt and drought stress, with the induction intensity decreasing as the stress duration prolonged. Interestingly, relatively high levels of up-regulation were observed in all time points of heat stress. In sweet potato, nearly all GR-RBPa genes in branch I showed significant suppression under various types of stress, while the two genes in branch II tended to be significantly induced by heat and prolonged osmotic stresses. Many GR-RBPa genes in tomato exhibited up-regulation either in the short or long term under the stresses applied in this study, except for Solyc01g109660.2 and Solyc10g051380.1, which were slightly down-regulated at all time points during cold stress treatment. It's important to note that Os07g0602600, GRMZM2G131167, and g7929 from branch II could not be appropriately detected, despite our attempts with different primers.

### AS patterns of GR-RBPa genes from different plants under different environmental conditions

**Figure 9:**
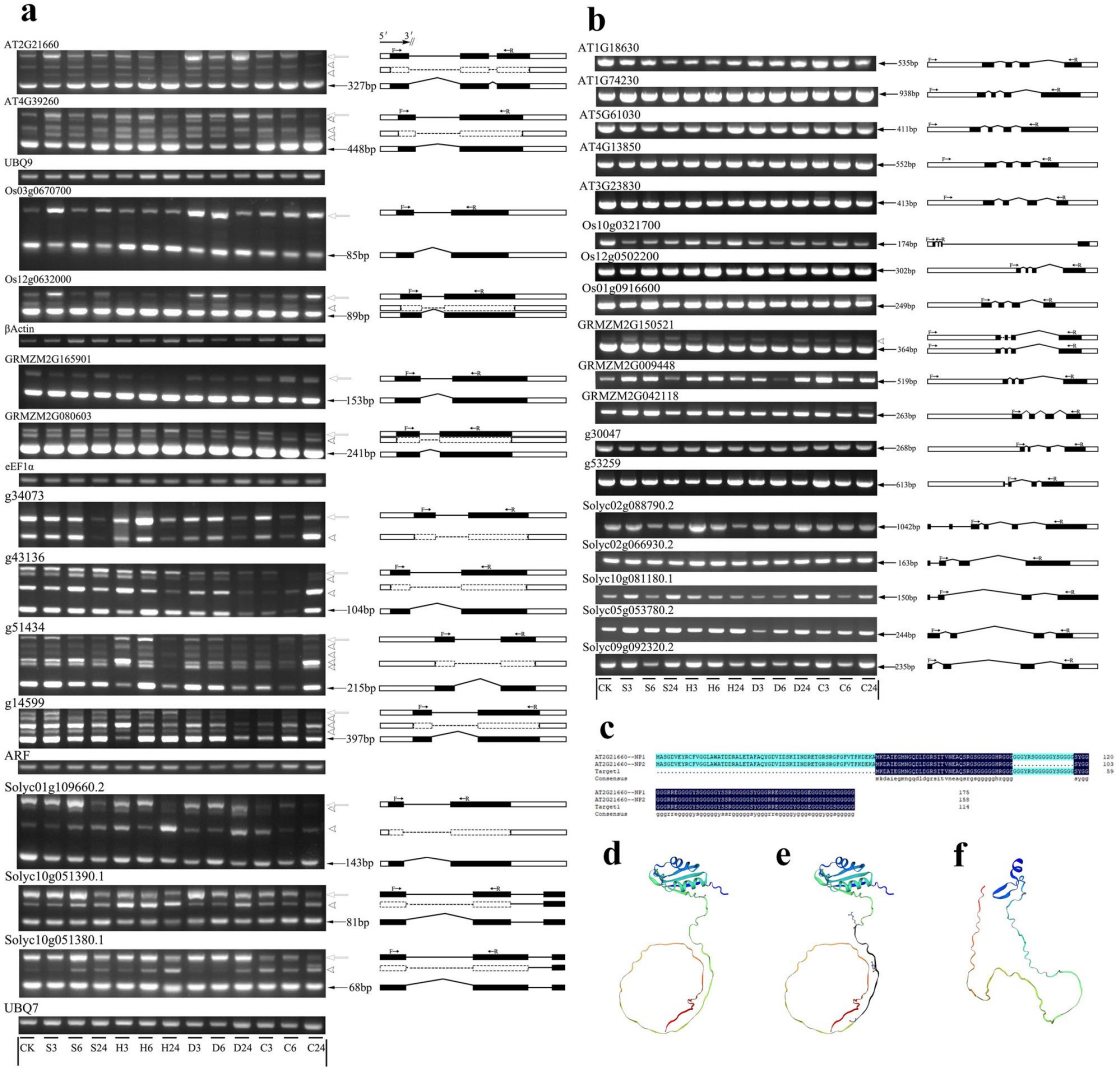
Splicing Patterns of Pre-mRNAs of GR-RBPa Genes and Potential Protein Variants. Splicing patterns of pre-mRNAs of GR-RBPa genes in branch I (**a**) and branch II (**b**). Key: The solid arrow marks the constitutively-spliced isoform, the hollow arrow marks the full intron retention isoform, and the triangular arrow marks other alternatively spliced isoforms. Gene names are presented above. Internal reference genes include UBQ9, βActin, EF1α, UBQ7, and ARF. The gene structure, binding sites of primers, and potential components of mRNA isoforms amplified in RT-PCR experiments are shown on the right. The solid black box represents the exon region. The straight line indicates the retained intron. The refraction line indicates the spliced intron. The hollow black box represents UTR. Dashed lines and dashed boxes stand for alternatively spliced intron and exon fragments with uncertain splice sites. (**c**–**f**) Predicted amino acid sequences and secondary structures of protein variants of the gene AT2g21660 in Arabidopsis. (**c**) The alignment of potential amino acid sequences resulting from alternative spliced mRNAs. (**d**) Secondary structure of the protein variant translated from the constitutively spliced mRNA isoform. (**e**, **f**) Potential secondary structures of protein variants translated from two alternatively spliced mRNA isoforms.

To further investigate the expression patterns of GR-RBPa genes at the post-transcriptional level, we utilized AS detection RT-PCR, a method previously established^45^. According to the results, all 13 genes in branch I underwent AS, even under normal conditions (Fig. 9a). For instance, two genes from Arabidopsis, AT2G21660 and AT4G39260, each produced 3 transcripts under normal conditions. Similarly, the two genes in rice, Os03g0670700 and Os12g063200, produced 2 and 3 transcripts, respectively. It is interesting to note that GR-RBPa genes from dicots generally produced more mRNA isoforms than those from monocots, suggesting a higher number of AS patterns in GR-RBPa genes from dicots.

AS patterns of all these GR-RBPa genes in branch I were altered by at least one kind of abiotic stresses. For example, the Arabidopsis gene AT2G21660 generated an additional mRNA isoform under salt, drought and heat conditions compared to the control, but produced only the constitutively-spliced isoform after 24 h of cold stress (C24). Moreover, stress-induced AS sometimes resulted not only in an increase or decrease in the number of isoforms but also in changes in the relative abundance of different isoforms. For example, there was a noticeable increase in the intensity of the fully intron-retained isoform of AT2G21660 after 3 h of salt stress treatment (S3). Similar phenomenon was found in the drought stress treatment, especially in D3 and D24. In contrast, the intensity of the fully intron-retained isoform decreased, while the ratios of the constitutively-spliced isoform and another alternatively-spliced isoform increased in H24. Extensive AS regulation was also found in another Arabidopsis gene, AT4G39260. The relative abundance of the constitutively-spliced isoform of this gene increased significantly in response to cold treatment but decreased noticeably under drought stress.

In the case of rice, there were noticeable up-regulations of the intron-retained isoforms of the two GR-RBPa genes in response to S3, D3, D6, and C24 treatments. However, the AS patterns of the two GR-RBPa genes from maize appeared to be less affected by stresses, except for GRMZM2G165901 under H6 and H24 conditions, and GRMZM2G080603 under H24 and C6 conditions. Additionally, significant stress-induced changes in splicing patterns were also observed in GR-RBPa genes from sweet potato and tomato. It's worth noting that, although changes in AS patterns of these genes occurred after different durations of stress treatments, the samples treated for 24 h usually exhibited the most significant disturbance in AS patterns when compared to the control sample.

It is interesting to note that the GR-RBPa genes in branch II were less alternatively spliced. Among the 18 GR-RBPa genes tested in branch II, only one gene (GRMZM2G150521 from maize) underwent AS to generate an additional isoform, which appeared to be induced by various types of stresses (Fig. 9b).

Components of alternatively spliced mRNA isoforms were predicted through their sizes and shown in right beside the gel panel (Fig. 9). Taking AT2G21600 as an example, the potential translation products of two AS mRNA isoforms were compared with that of the constitutively isoform (Fig. 9c). The amino acid (AA) sequences generated form AS mRNA isoforms were truncated proteins, one lacking a 17 AA fragment from the glycine-rich region with less effect on the 3D structure of the protein, while the other lacked a part of the AA fragment from the N-terminal (Fig. 9d–f).

## Discussion

Glycine-Rich RNA-Binding Proteins (GR-RBPs) are ubiquitous in plants and play vital roles in plant growth and stress responses. These proteins were initially discovered in petunia^48^, and subsequent studies have identified them in various plant species, including Arabidopsis^49^, *Physcomitrella patens*^50^, rice^51^, rapeseed (*Brassica napus*)^52^, maize^23^, *Ipomoea trifida*^21^, and Chinese cabbage (*Brassica rapa*)^53^. In our investigation, we identified 7 and 8 GR-RBPa subclass genes in sweet potato (*Ipomoea batatas*) and tomato (*Solanum lycopersicum*), respectively. The number of GR-RBPa genes in these two plant species remained relatively stable compared to Arabidopsis, rice, and maize^24^, indicating a consistent proliferation rate of GR-RBPa genes in both monocot and dicot plant species. Furthermore, the consistent proliferation rate of gene members of GR-RBPa proteins across different plants during evolution suggests their essential roles as housekeeping genes in plants^54^.

The GR-RBPa protein is chaeaterized by a conserved RNA Recognition Motif (RRM) domain located at the N-terminal and a glycine-rich C-terminal. Both terminals of GR-RBPa proteins play important roles in their functionality. Studies have shown that variations in the length of the glycine-rich region of AT3G223830 can lead to functional differences in cold stress adaptation, while another study suggested that the difference may originate from the N-terminal RRM domain itself^55,56^. Our analysis revealed significant variation in the length of the C-terminal region among different GR-RBPa members (Fig. 2). Moreover, GR-RBPa proteins from the five plant species could be further classified into two branches based on the conserved motifs they possess, consistent with previous findings^24^. For instance, motif 8 is exclusively found in branch I, while motifs 4, 7, and 9 are unique to branch II. GR-RBPa proteins within the same branch of a plant species are believed to exhibit more conservative molecular functions than those between different branches. For example, AT2G21660 and AT4G39260 of branch I regulate their own pre-mRNAs, cross-regulate each other’s pre-mRNAs, and share several downstream pre-mRNAs^33,57^. In contrast, although both AT4G13850 (belonging to branch II) and AT2G21660 have been shown to enhance cold resistance in plants, AT4G13850 promotes the germination of Arabidopsis seeds under salt and drought stresses, while AT2G21660 has a negative effect on seed germination and seedling growth under salt or dehydration stress conditions^27,55,58,59^. In addition, overexpression of AT3G23830 in branch II did not confer cold tolerance but only enhanced salt and drought resistance in the transgenic plants, similar to AT4G13850^56^. Interestingly, our protein–protein interaction analysis revealed that GR-RBPa proteins in branch I shared more interacting proteins than those in branch II, suggesting more conserved molecular function of GR-RBPa proteins in branch I (Fig. 3, Supplemental Fig. 2). Multiple and diverse roles of GR-RBPa proteins could be attributed to the different motifs they possess, as protein–protein interactions and binding of specific nucleotides of proteins may be affected by the motifs they contain^60^. Therefore, further systematic comparisons are needed in the future to confirm the functions of specific motifs of GR RBPa proteins.

GR-RBP proteins are known to play crucial roles in regulating plant growth and responding to stress^17,31^. Our analysis revealed that the promoters and introns of GR-RBPa genes are enriched with various cis-elements, including those involved in light signaling, hormone signaling, growth and development, and stress responses (Fig. 5). This indicates that GR-RBPa genes are subject to regulatory control at the expression level in response to various internal and external cues. Previous studies have shown that GR-RBP genes are highly induced by heat stress in sorghum^61^ and *Pinellia ternate*^62^. On the other hand, the expression level of *LpGRP1* transcript in ryegrass was significantly induced with prolonged exposure to cold stress^63^. Additionally, the transcriptional levels of PpGRPs in *Physcomitrella patents* fluctuated during prolonged cold stress^50^. In our study, to ease and ensure the comparability between individual investigations, we use the same hydroponics-based experimental conditions when cultivate different plants. However, it is important to note that this approach may introduce extra stresses, such as oxygen and soil microbial deficiency, to the plants^64^. It's worth mentioning that we did not calculate whether there are differences and how significant they are in the gene transcription and splicing patterns of GR-RBP genes between plants cultivated in soils and those grown in water, as our focus was primarily on the effects generated from salt, drought and temperature here.

When we subjected the five plant species to salt, drought and temperature stresses, we observed significant and intricate changes in the expression of GR-RBPa genes compared to their expression levels in the original culture solution. For example, most GR-RBPa genes from maize and tomato showed transcriptional induction in response to all types of abiotic stress employed. Conversely, GR-RBPa genes in rice tended to be down-regulated under salt and cold stresses. Additionally, GR-RBPa genes in branch I were generally downregulated by all kinds of stress treatments, while those in branch II were more frequently induced by stress. The expression of some GR-RBPa genes exhibited fluctuations in response to different types of abiotic stress or even different durations of a particular stress. Moreover, homologous GR-RBPa genes from the same plant species could be regulated in opposite directions by the same stress. However, transcriptional regulations of GR-RBPa genes did not exhibit clear divergence between monocots and dicots. In conclusion, transcriptional levels of these GR-RBPa genes are not conserved among the plant species we tested. Therefore, it is essential to investigate in future studies whether transcriptional regulation of the GR-RBPa genes is controlled by coordinated modulations between different cis-elements. Alternatively, there could be other unidentified cis-elements that significantly affect transcriptional levels.

Accumulating studies have demonstrated that pre-mRNA alternative splicing (AS) regulation plays essential roles in plant growth and stress response because alterations in the relative abundances of different transcript isoforms can affect the abundance and diversity of protein products^65–68^. In our study, the results of gene structure analysis showed a clear distinction in the number of introns between GR-RBPa genes in branch I and branch II. The genes in branch I have fewer introns (only 1 or 2) than those in branch II (Fig. 4), suggesting a potential co-evolution of gene structure and protein function among GR-RBPa proteins in plants. Surprisingly, RT-PCR results demonstrated that all of the GR-RBPa genes in branch I tested were found to be alternatively spliced and subsequently generated multiple mRNA isoforms, while nearly all GR-RBPa genes in branch II were only constitutively spliced, even though they contained more introns (Fig. 9). In addition, AS patterns changed significantly among GR-RBPa genes in branch I, for various types of stress or different durations of the same stress treatment could induce different AS variations in pre-mRNAs of these genes. Moreover, AS regulations of homologous GR-RBPa genes changed significantly among different plant species, and no clear divergence of AS regulation model was found between monocot and dicot species. These findings highlight the complexity and variability of AS regulations in coding genes of the RNA binding proteins (RBPs), which have also been recently reported in another family, serine/arginine-rich (SR) protein^45,69^.

The reason for the differential response of different branches of GR-RBPa genes to identical stress at both transcription and splicing levels remains unclear. It remains uncertain whether or not the evolution in gene structure results in specific regulation of the master genes themselves, and subsequently affecting downstream genes at the genome-wide level. Studies in different plants have shown that AS of pre-mRNA of GR-RBPa proteins can alter the splicing patterns of downstream genes^57,70^. In our experiments, there were also significant changes in the abundances of different AS transcripts of GR-RBPa proteins when the plants were subjected to environmental stresses. In-silico analysis has also indicated that potential truncated proteins may be generated through AS of AT2g21660 gene (Fig. 9c–f). Existing evidence supports that AS-induced protein variants have different functions in response to environmental changes. For instance, HAB1 protein variants generated through pre-mRNA AS regulation play opposite roles in ABA signaling^71,72^. Additionally, two protein variants of OsZIP1 generated through pre-mRNA AS regulate development of rice plant under light and dark conditions, respectively^73^. However, further functional studies are required to confirm how different GR-RBPa alternatively spliced variants affect the stress response and adaptation in plants.

In conclusion, the intricate and diverse expression regulation of GR-RBPa genes across different plant species, at both transcriptional and pre-mRNA splicing levels, prompts questions about the contribution of these regulatory mechanisms to plant development and stress adaptation. Despite sharing relatively conserved functional domains, the impact of their diverse expression patterns on growth and development of the plants remains unclear. Understanding the biological significance of the variations in each pre-mRNA isoform could be crucial for future application of this gene family in breeding crops for enhanced stress tolerance.

### Supplementary Information

Supplementary Figure 1.

Supplementary Figure 2.

Supplementary Figure 3.

Supplementary Information.

Supplementary Table 1.

Supplementary Table 2.

Supplementary Table 3.

Supplementary Table 4.

Supplementary Table 5.

Supplementary Table 6.

Supplementary Table 7.

## Data Availability

All main data of the study appear in the submitted article. Supplementary data are available online.
