# Supplementary figures and images for: Divergence in regulatory mechanisms of GR-RBP genes in different plants under abiotic stress
Source: Sci Rep. 2024 Apr 16;14:8743. doi: 10.1038/s41598-024-59341-8 (PMC11021534; doi:10.1038/s41598-024-59341-8)

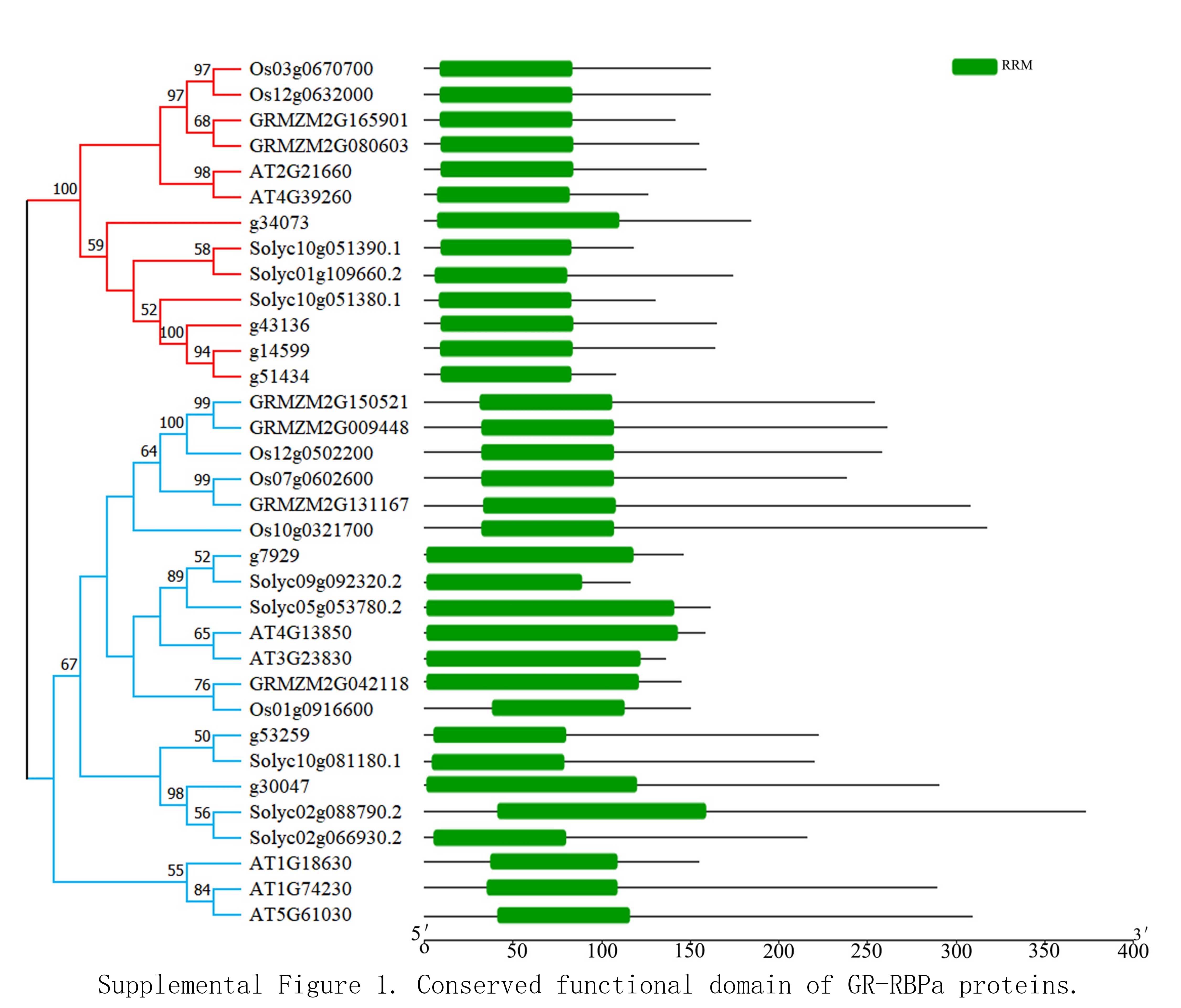

Supplement: Supplementary file 1 — Supplementary Figure 1. [file 41598_2024_59341_MOESM1_ESM.jpg]

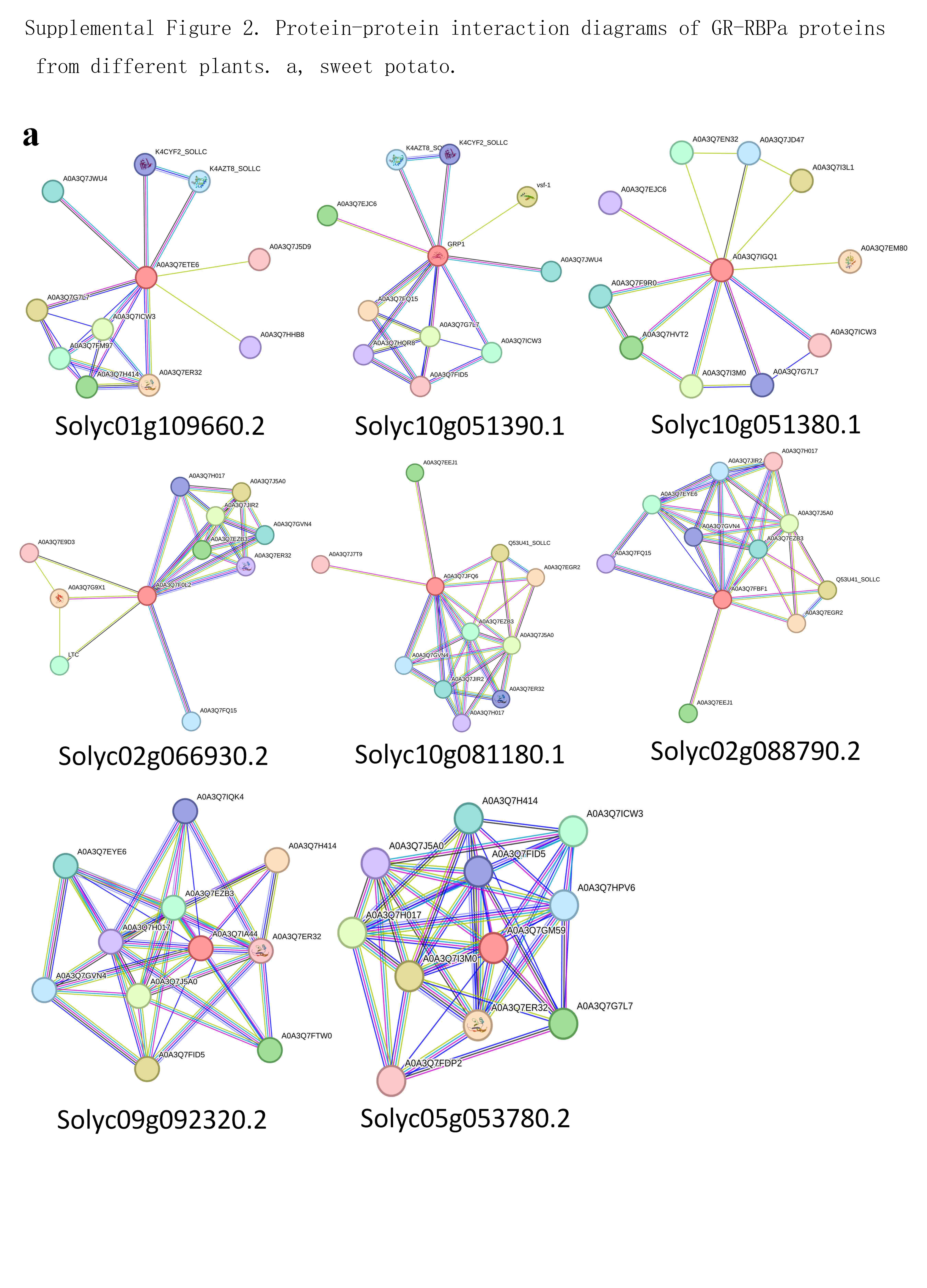

Supplement: Supplementary file 2 — Supplementary Figure 2. [file 41598_2024_59341_MOESM2_ESM.jpg]

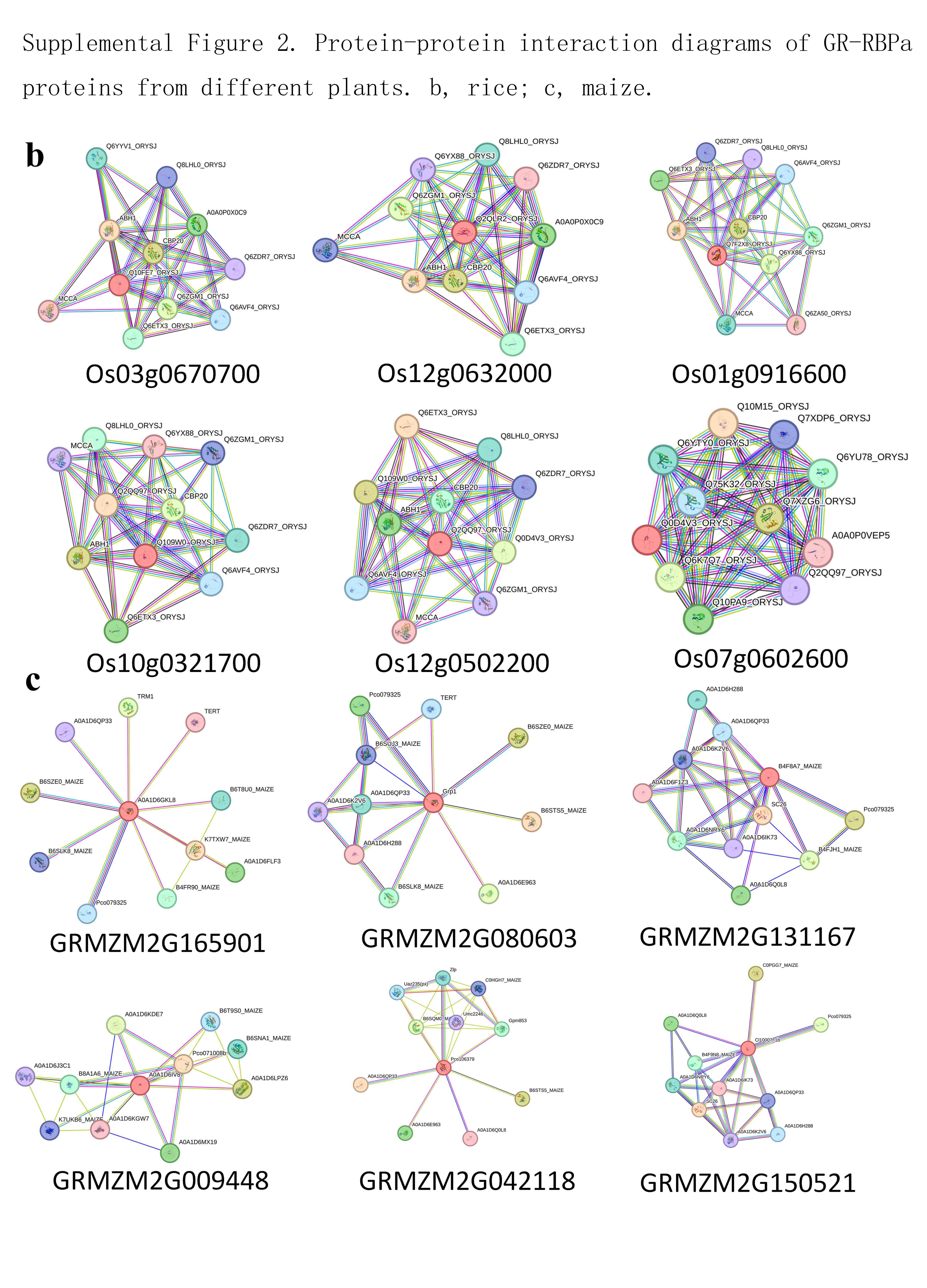

Supplement: Supplementary file 3 — Supplementary Figure 3. [file 41598_2024_59341_MOESM3_ESM.jpg]
