# Supplementary material for: Divergence in regulatory mechanisms of GR-RBP genes in different plants under abiotic stress
Source: Sci Rep. 2024 Apr 16;14:8743. doi: 10.1038/s41598-024-59341-8 (PMC11021534; doi:10.1038/s41598-024-59341-8)
Supplement: Supplementary file 4 — Supplementary Information. [file 41598_2024_59341_MOESM4_ESM.pptx]

## Slide 1
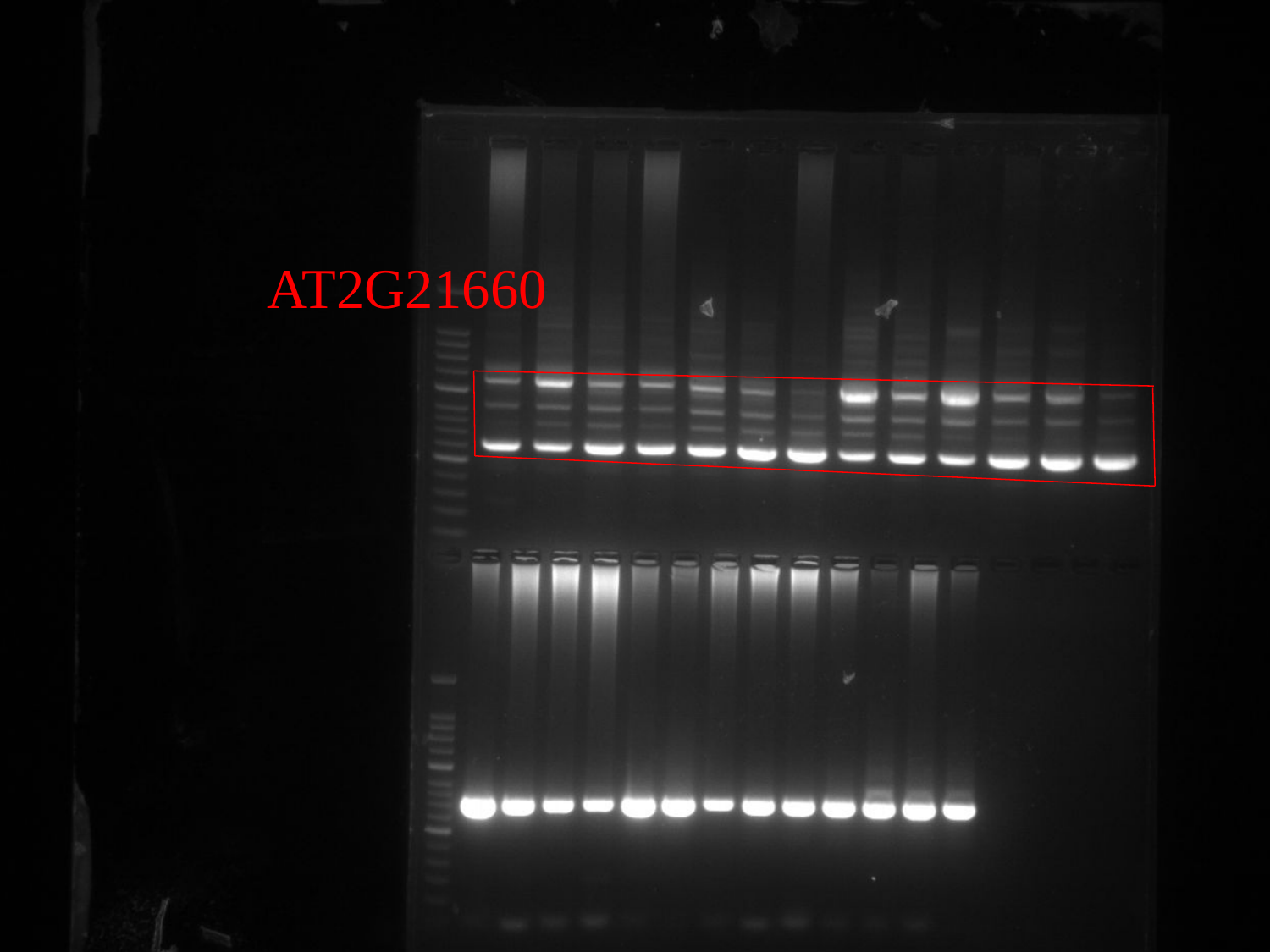

AT2G21660

## Slide 2
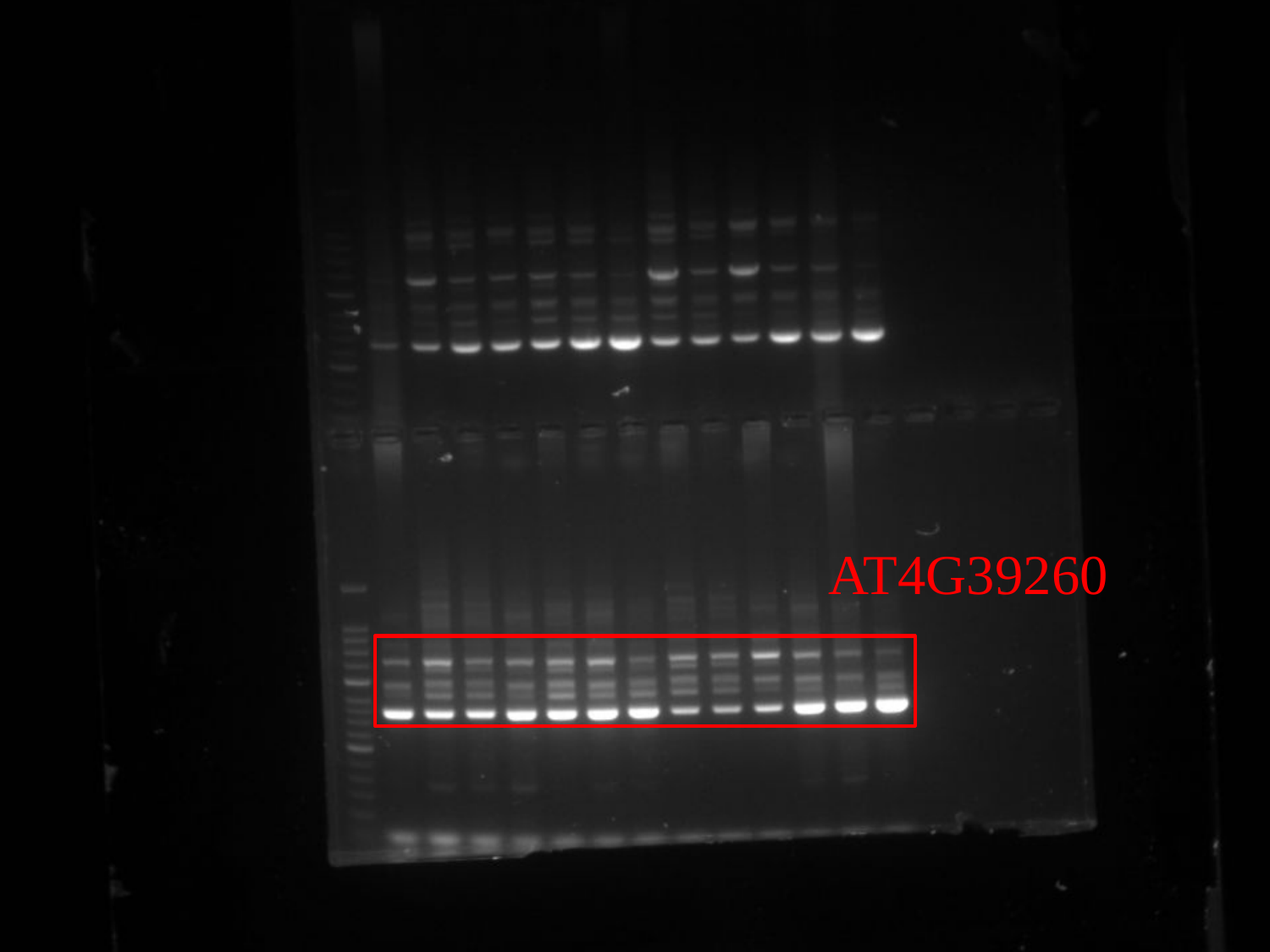

AT4G39260

## Slide 3
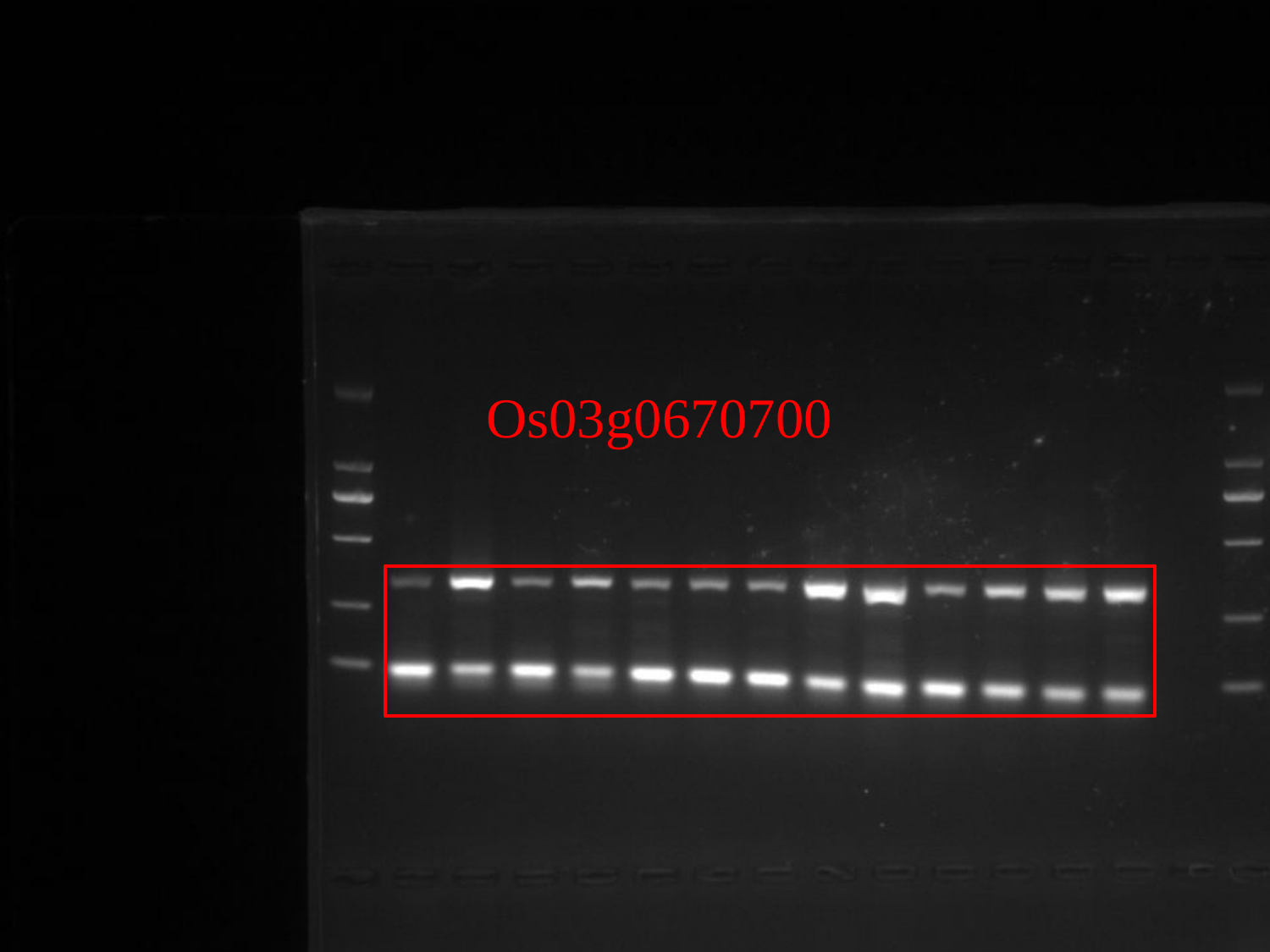

Os03g0670700

## Slide 4
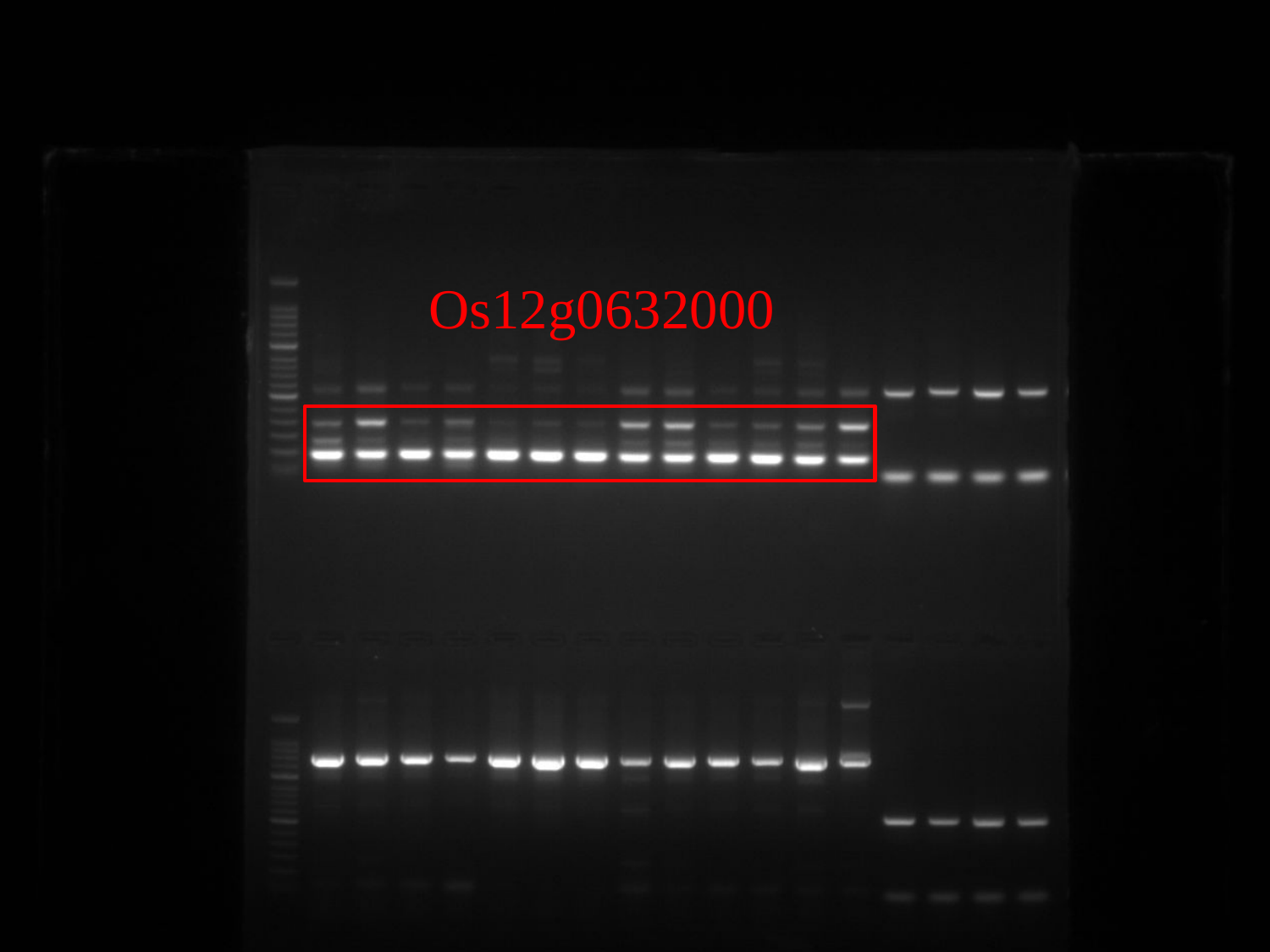

Os12g0632000

## Slide 5
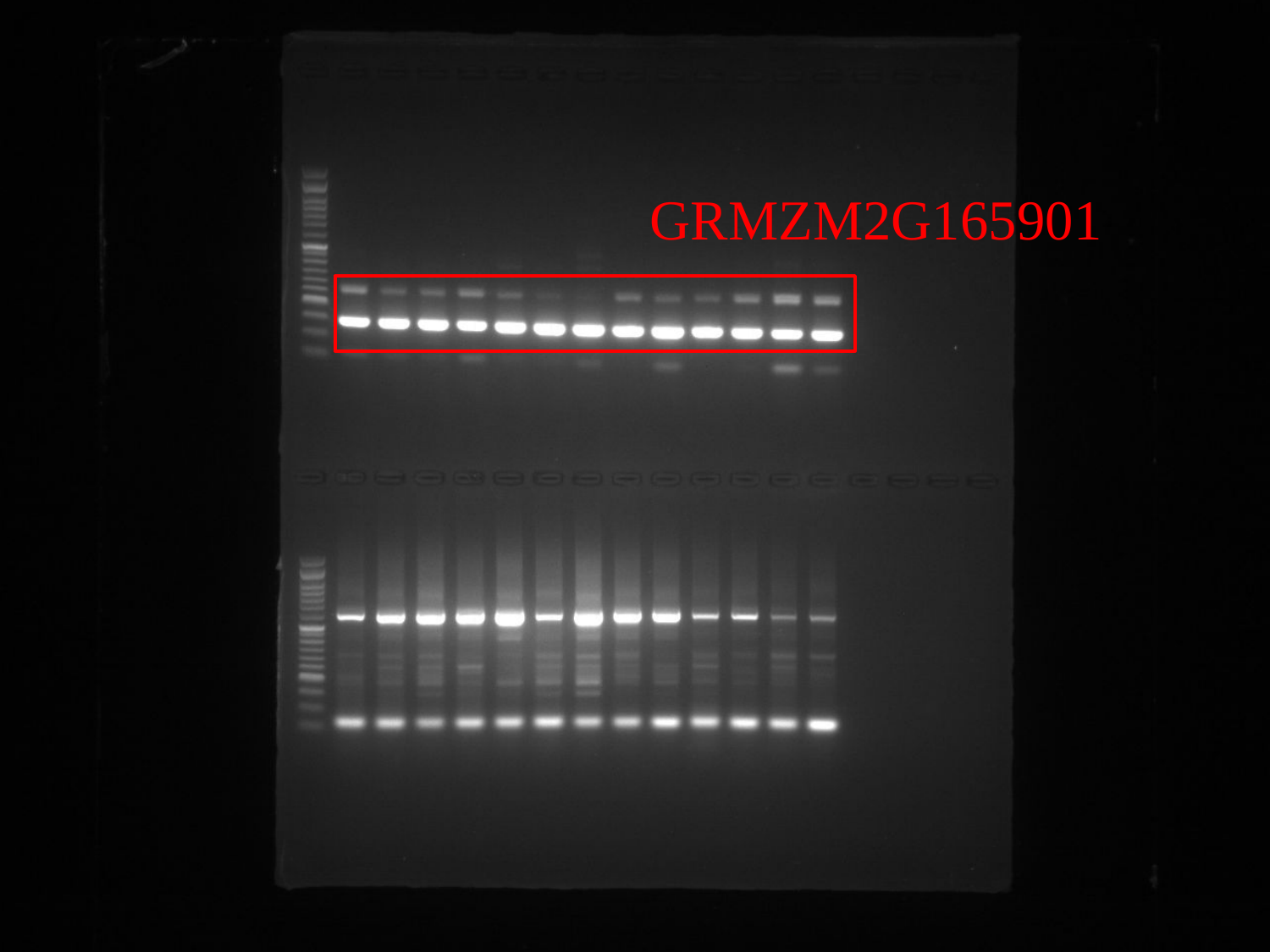

GRMZM2G165901

## Slide 6
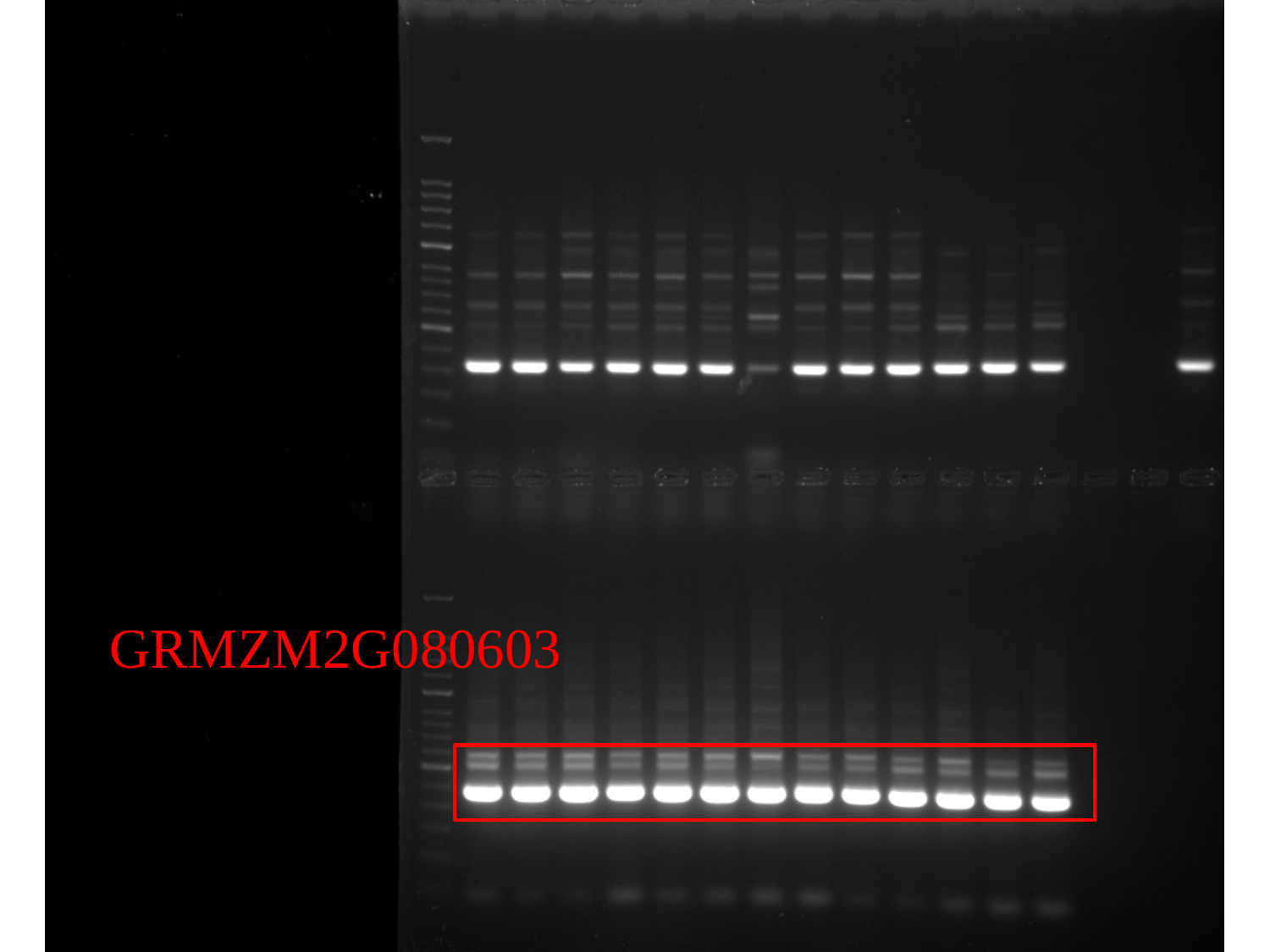

GRMZM2G080603

## Slide 7
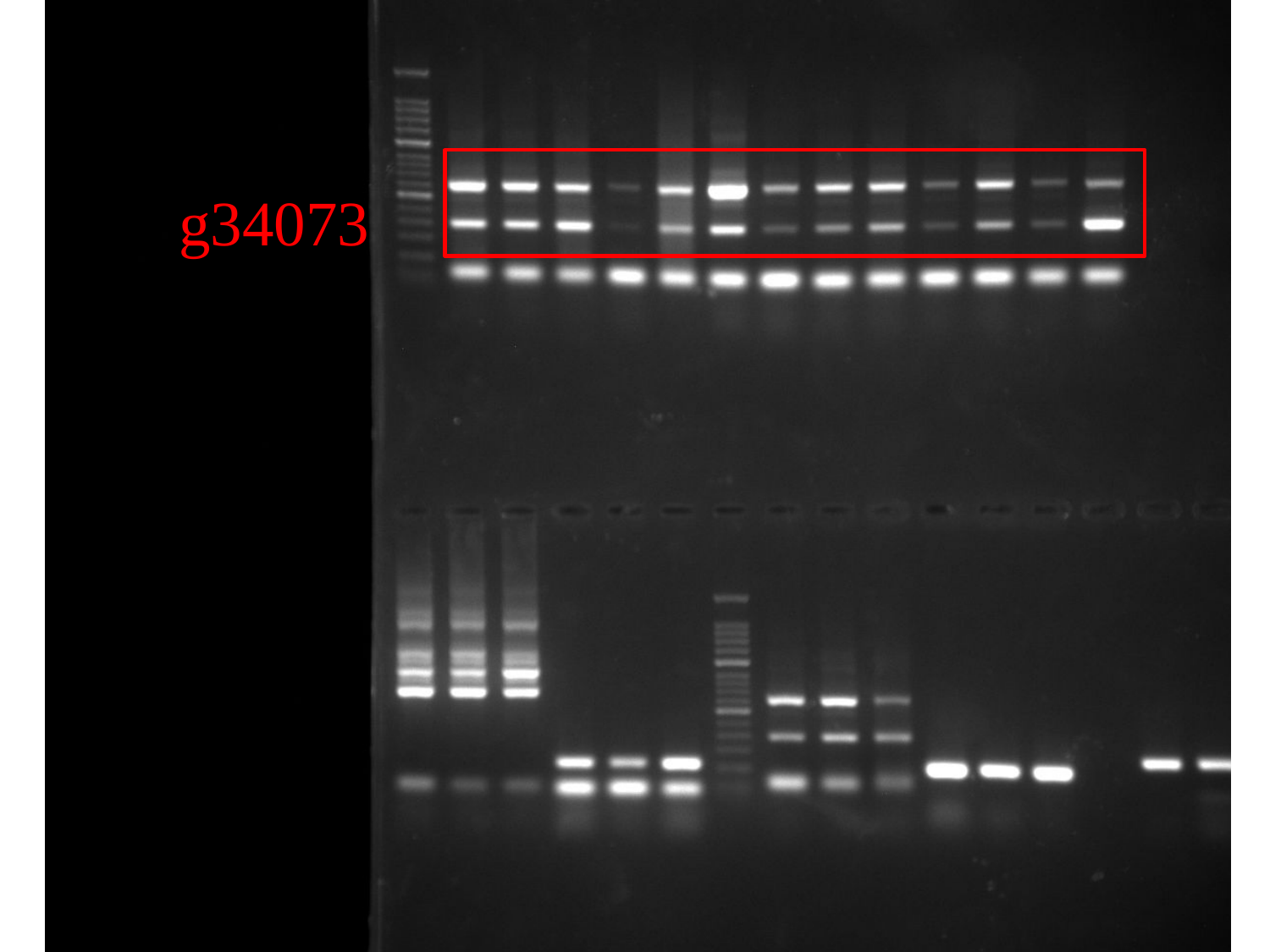

g34073

## Slide 8
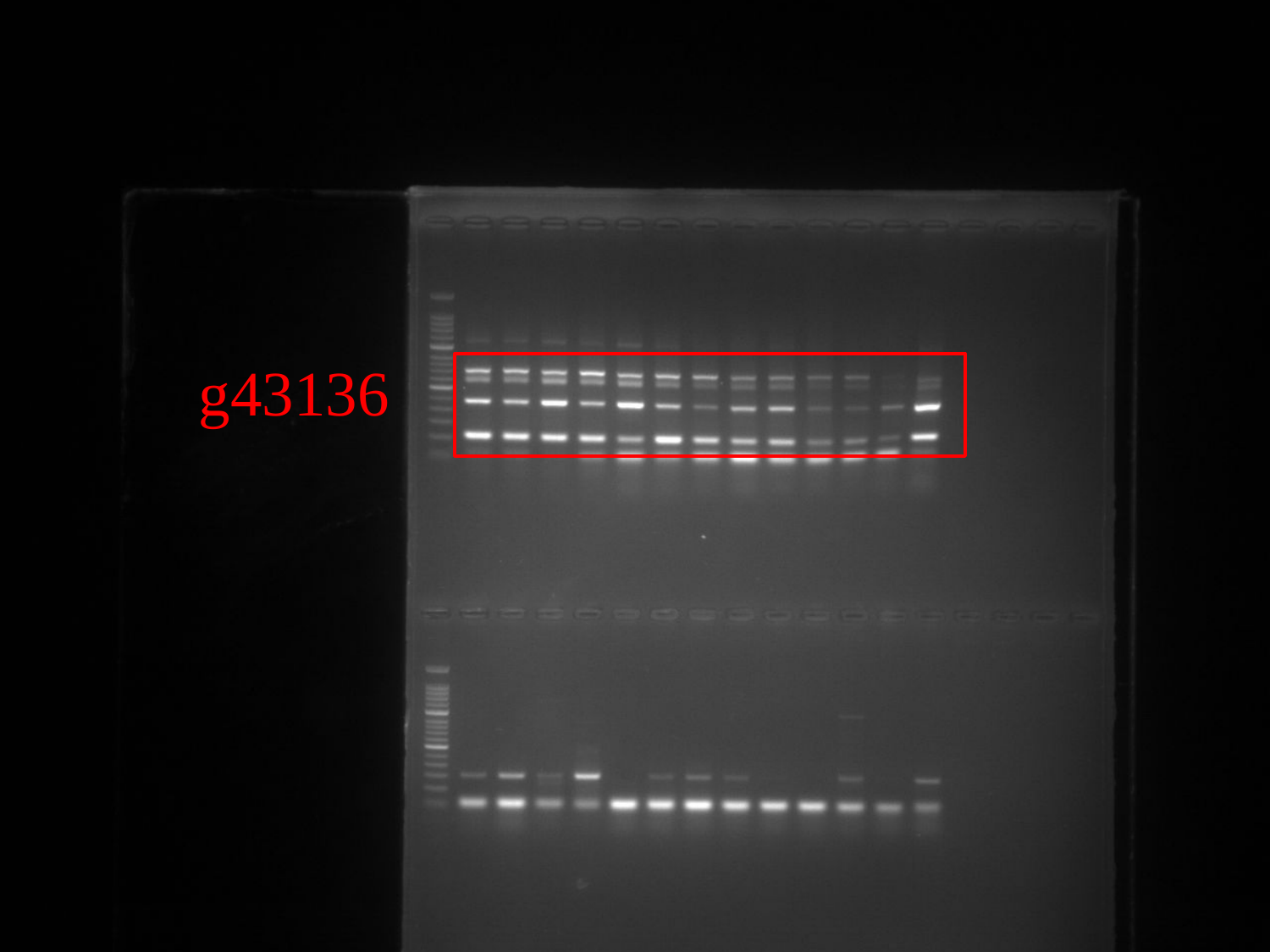

g43136

## Slide 9
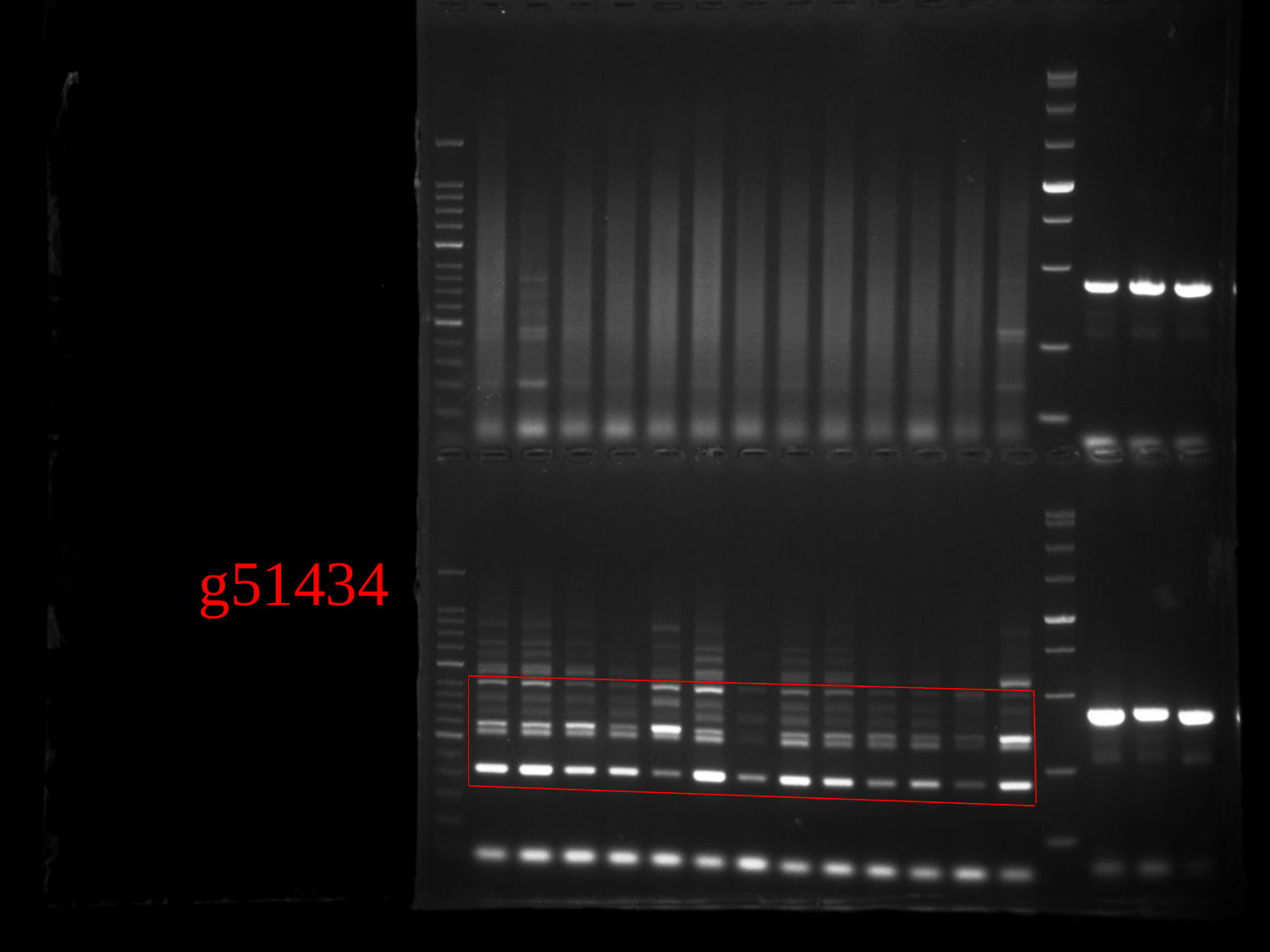

g51434

## Slide 10
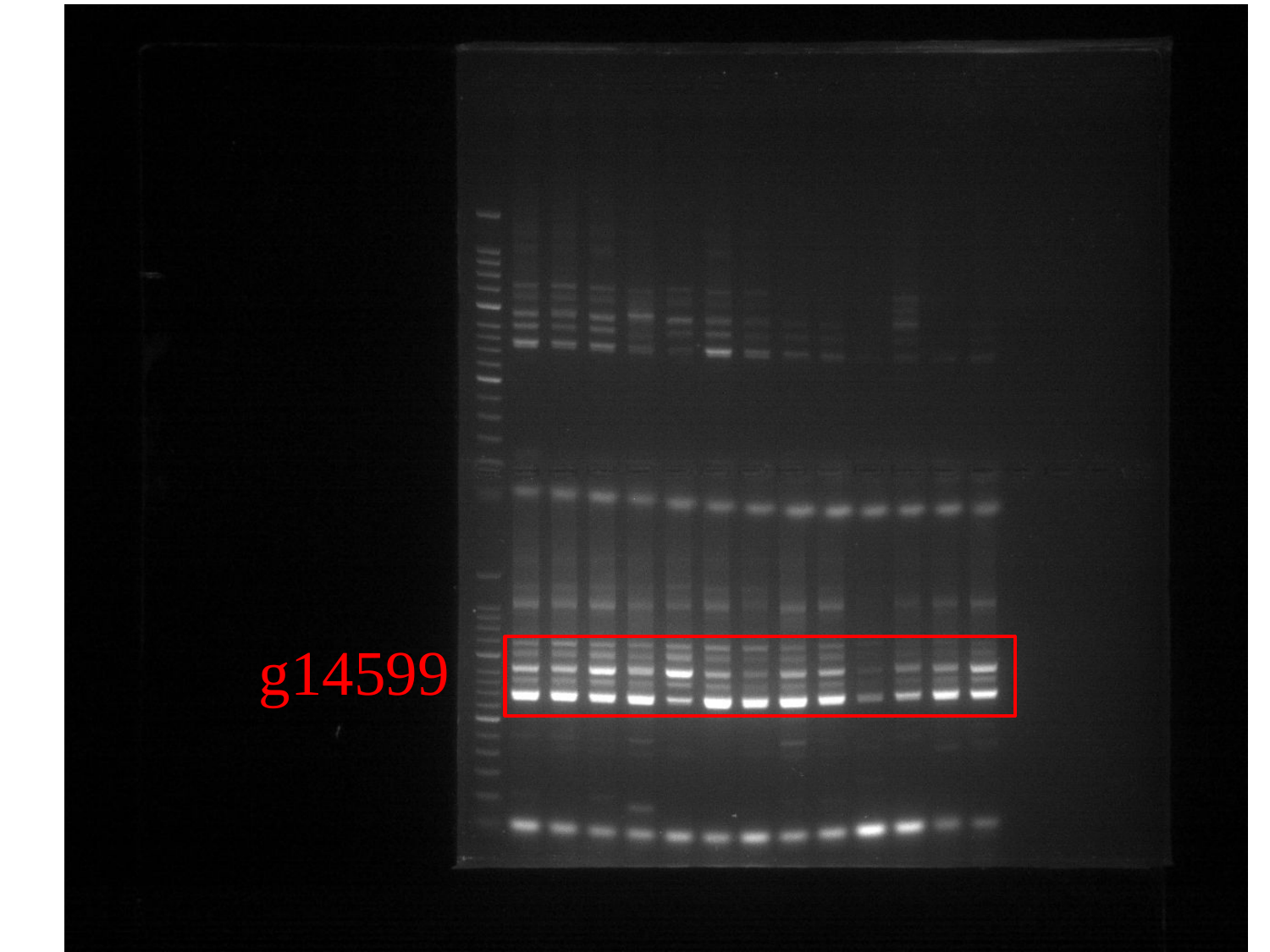

g14599

## Slide 11
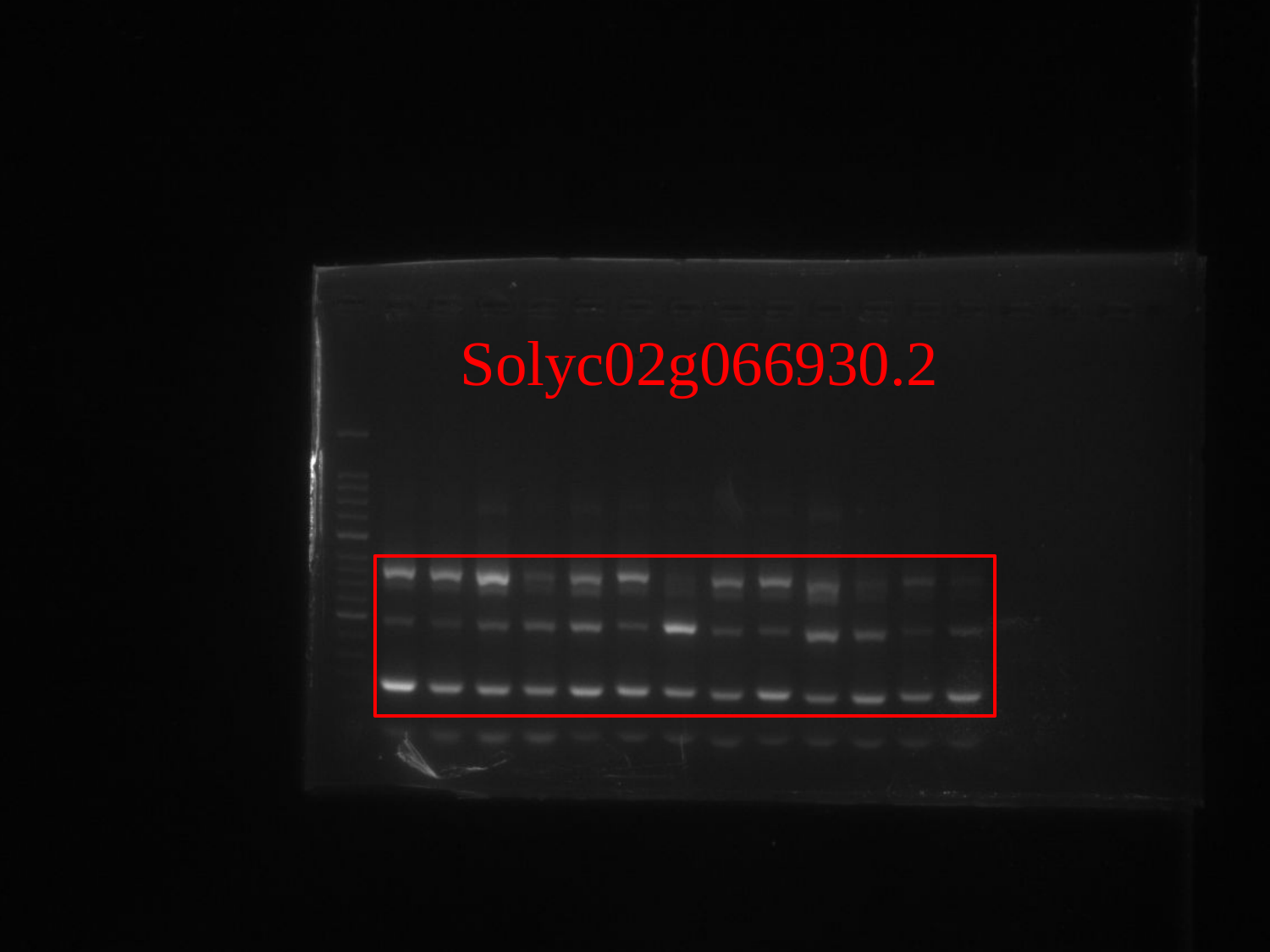

Solyc02g066930.2

## Slide 12
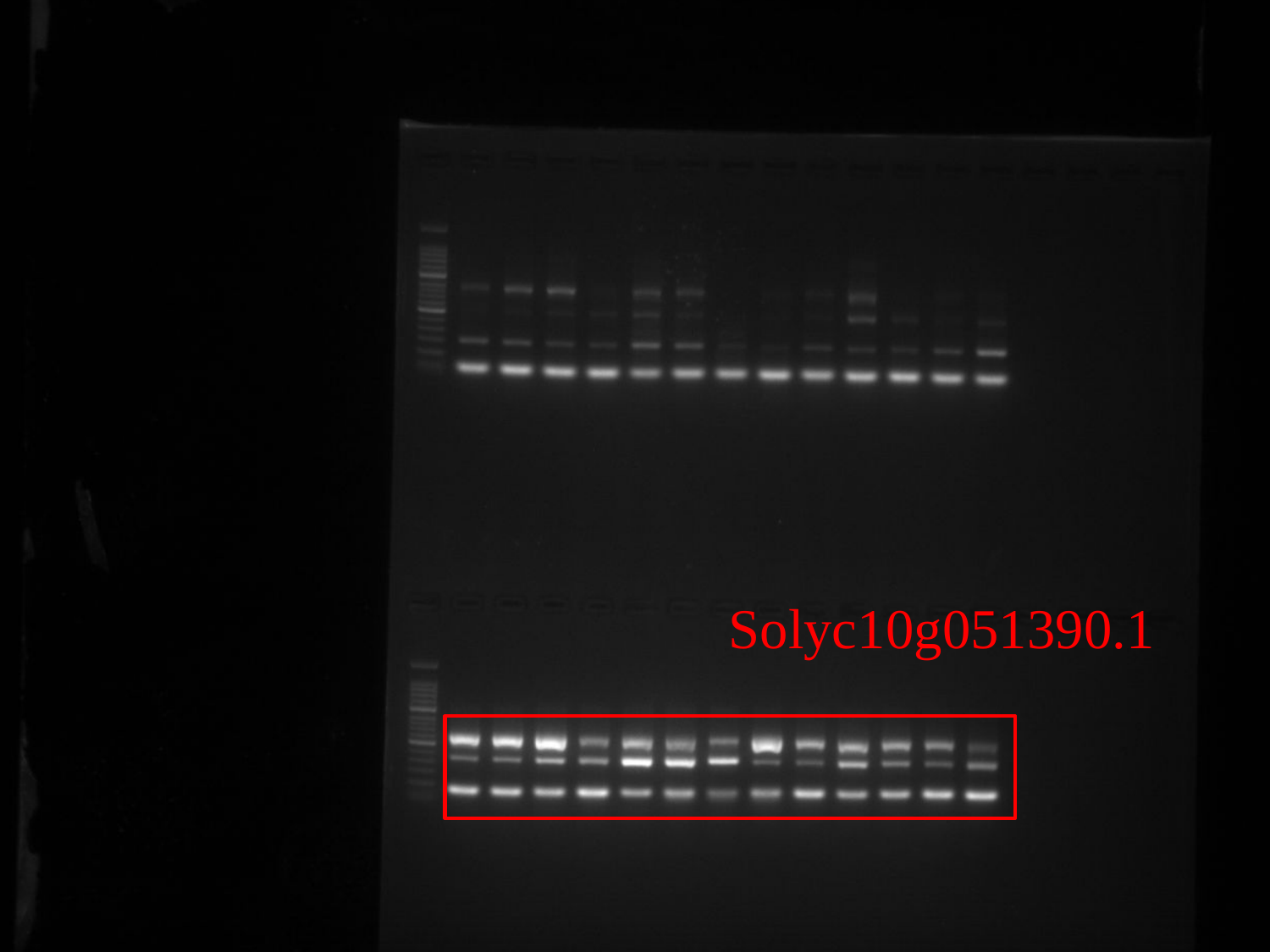

Solyc10g051390.1

## Slide 13
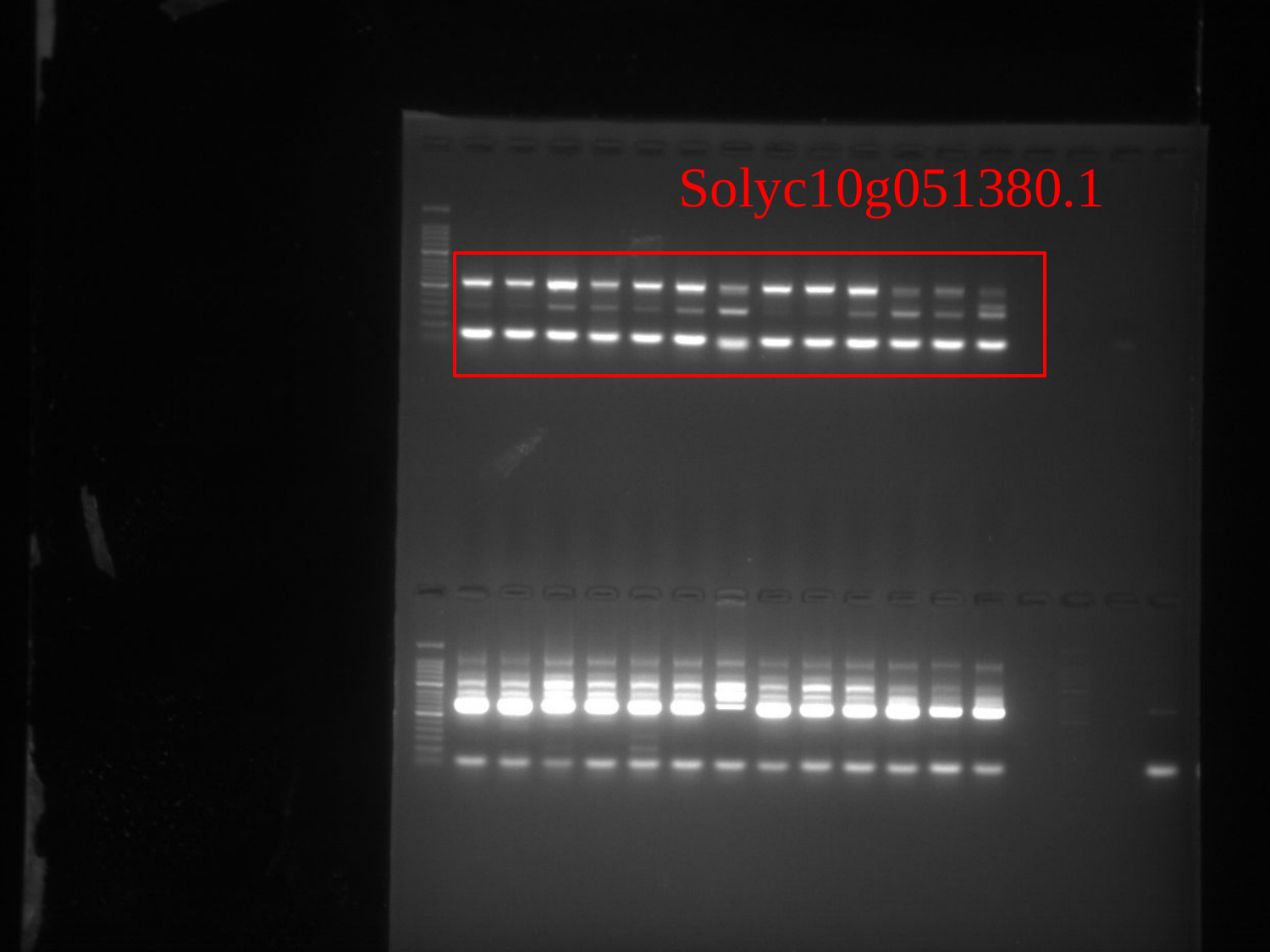

Solyc10g051380.1

## Slide 14
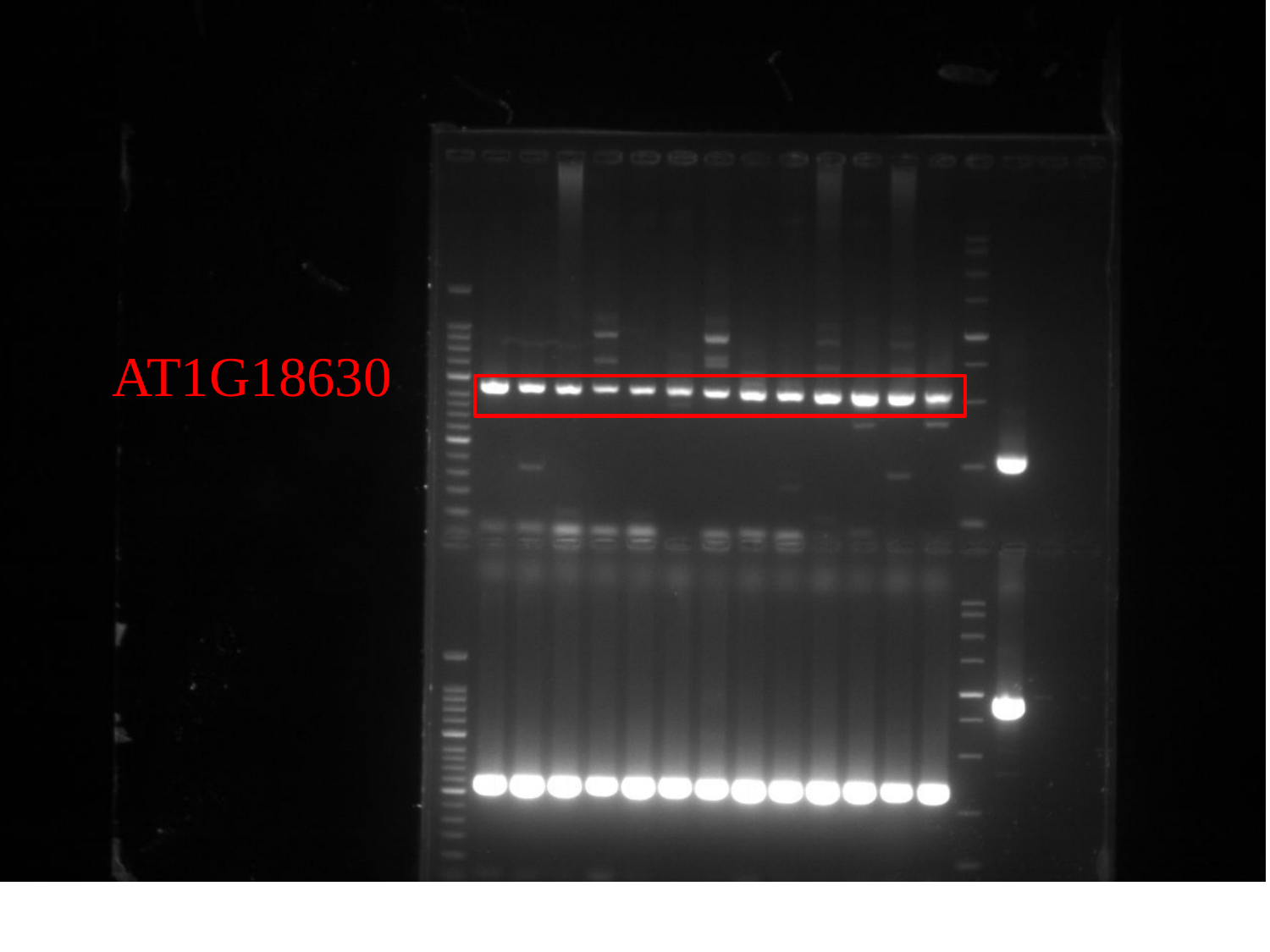

AT1G18630

## Slide 15
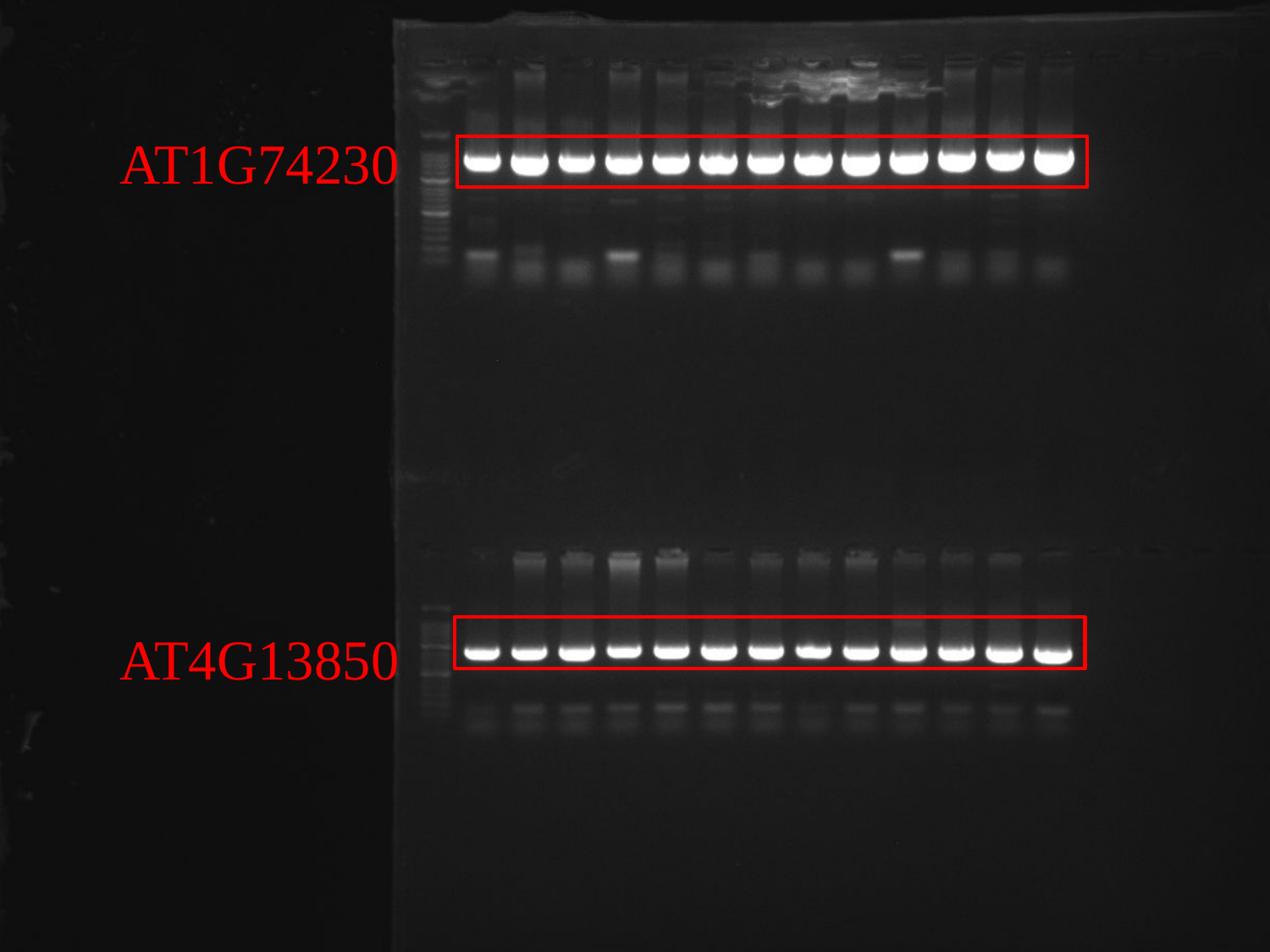

AT1G74230
AT4G13850

## Slide 16
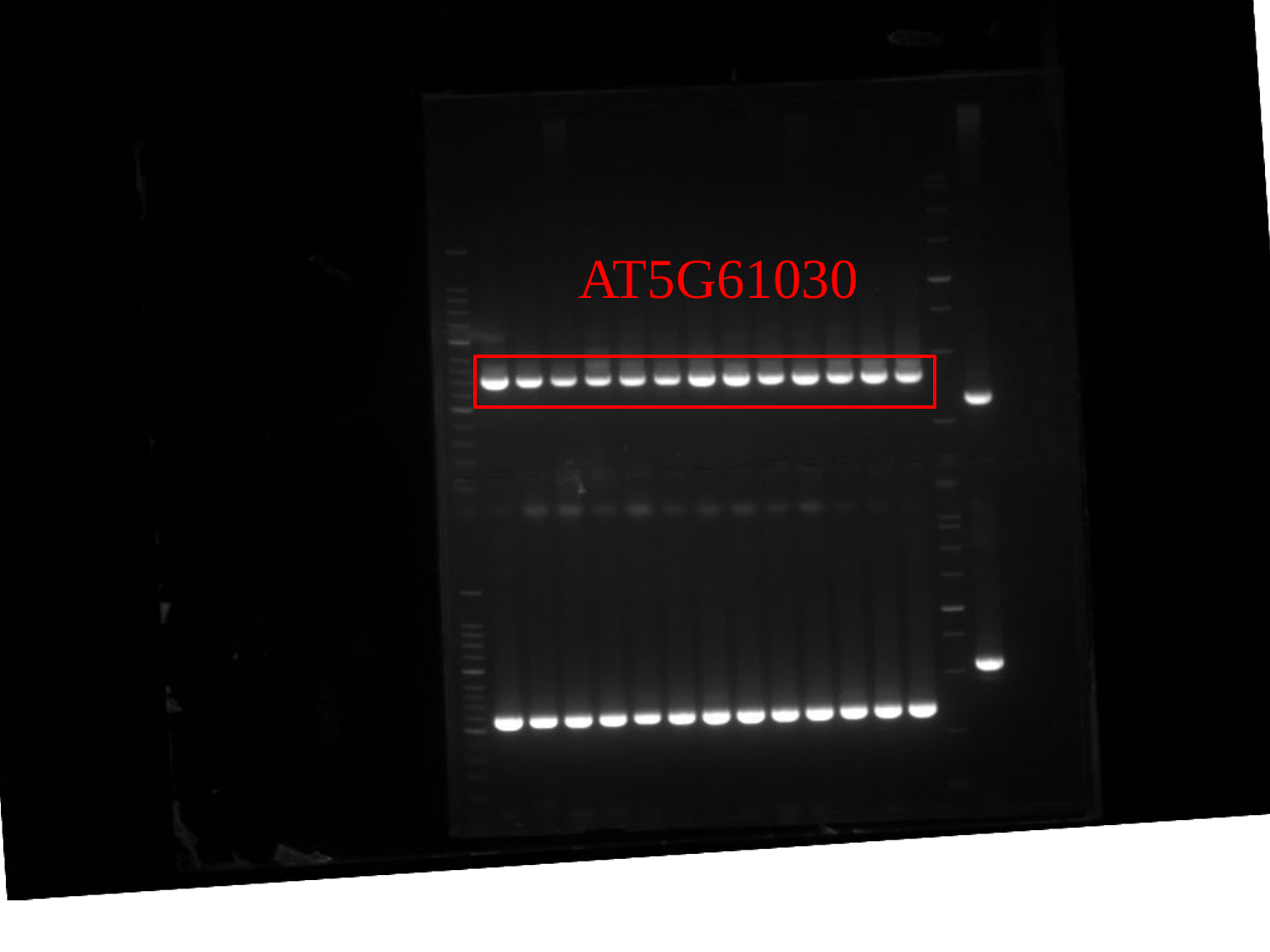

AT5G61030

## Slide 17
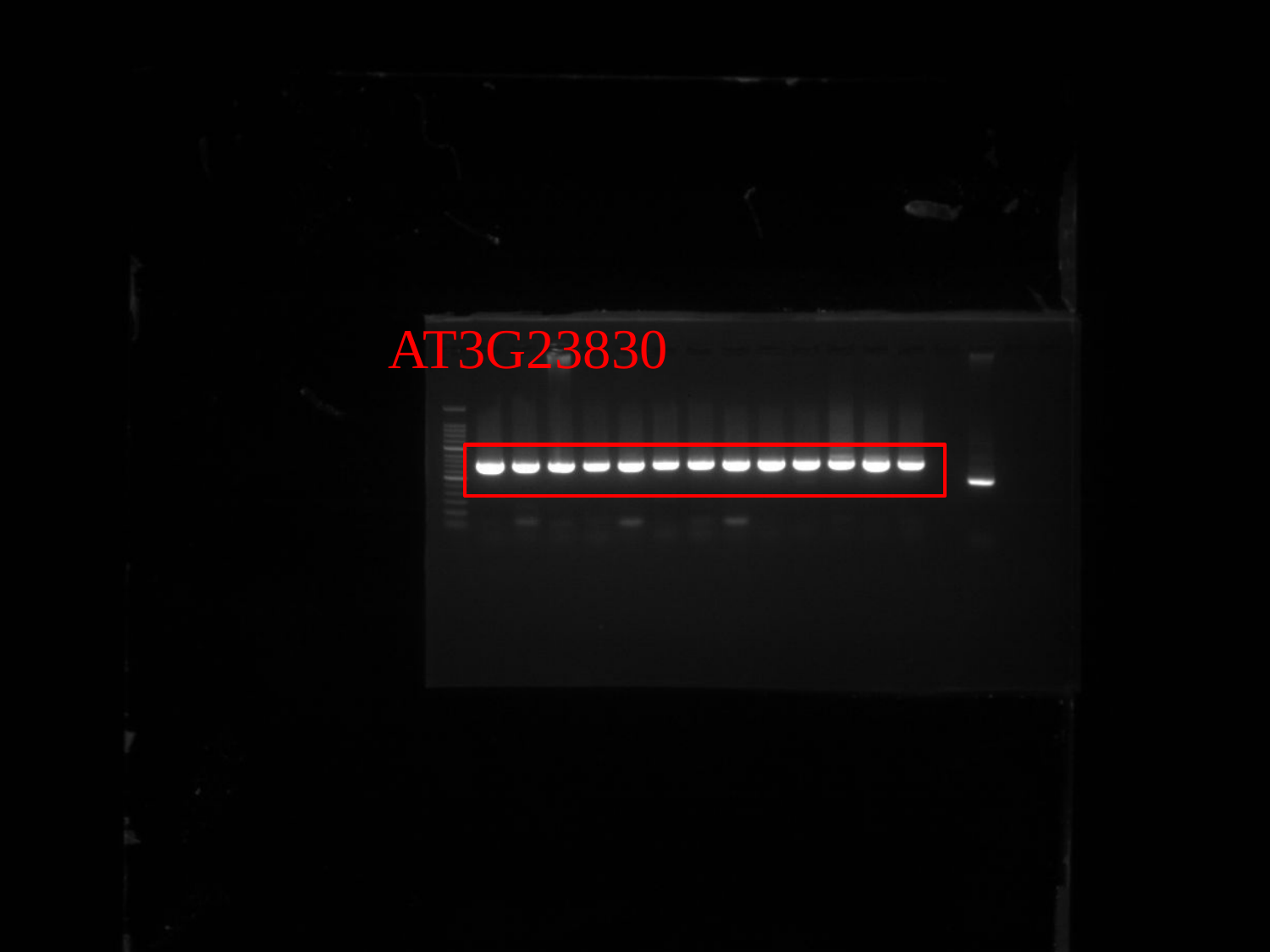

AT3G23830

## Slide 18
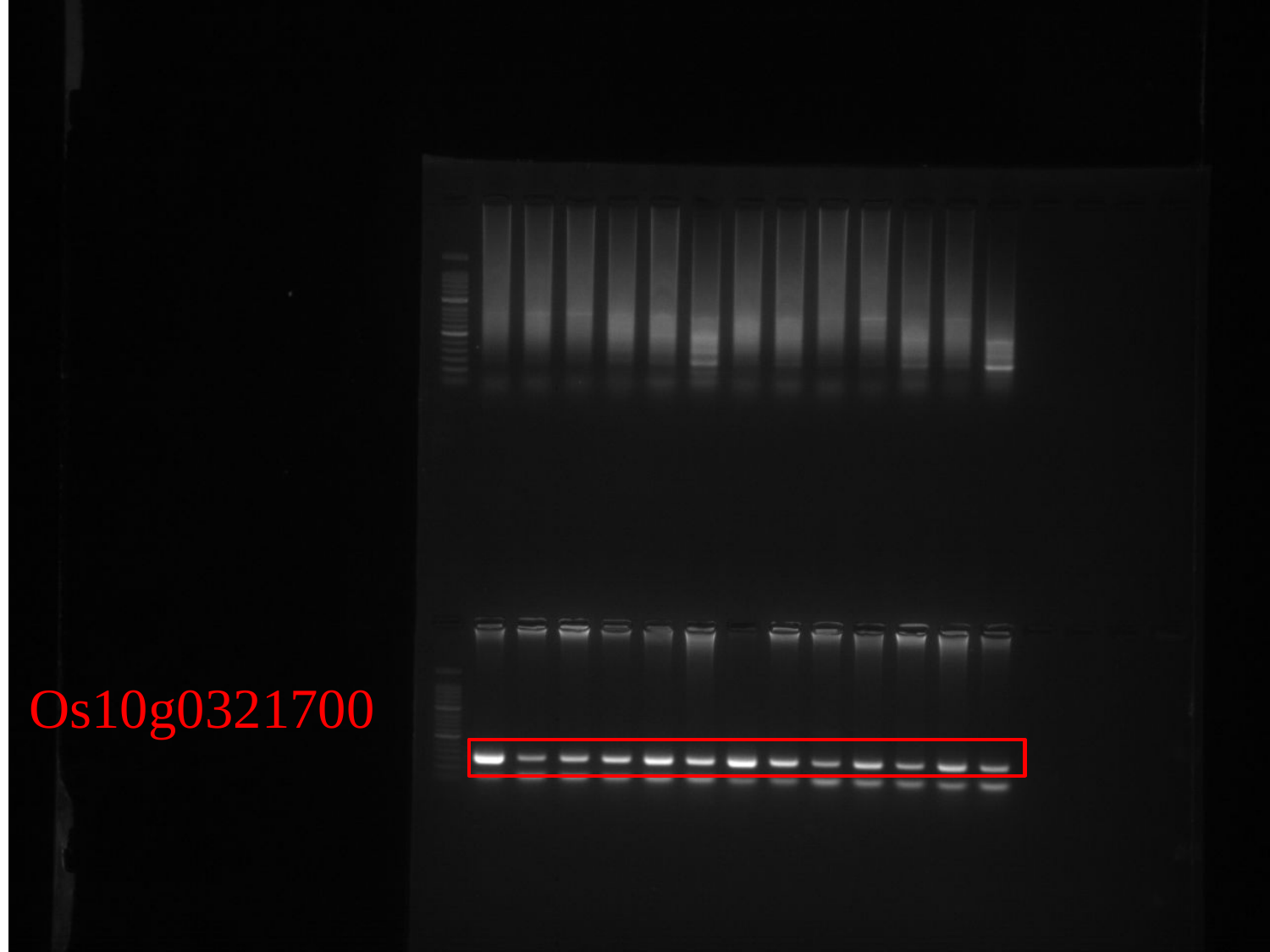

Os10g0321700

## Slide 19
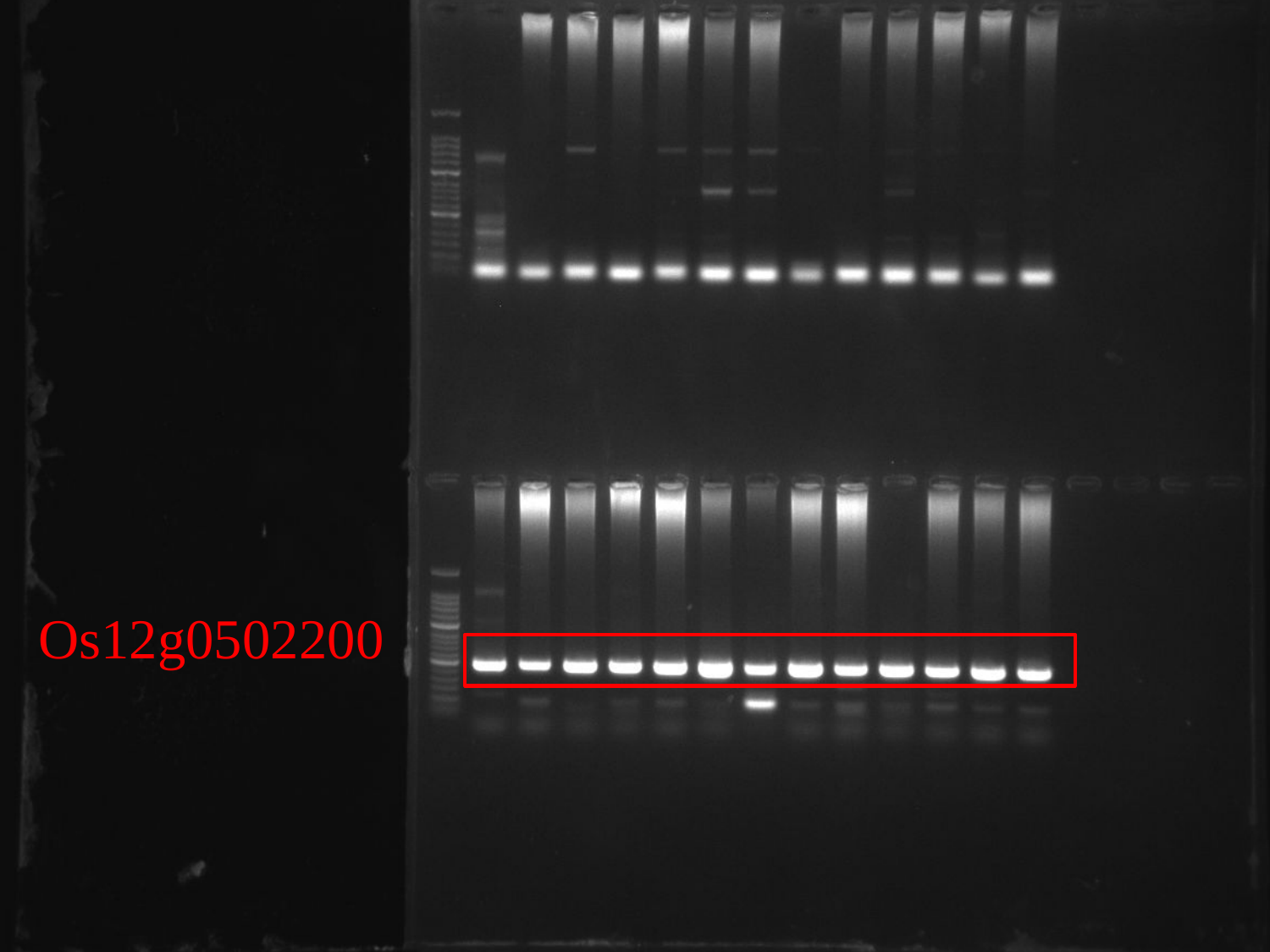

Os12g0502200

## Slide 20
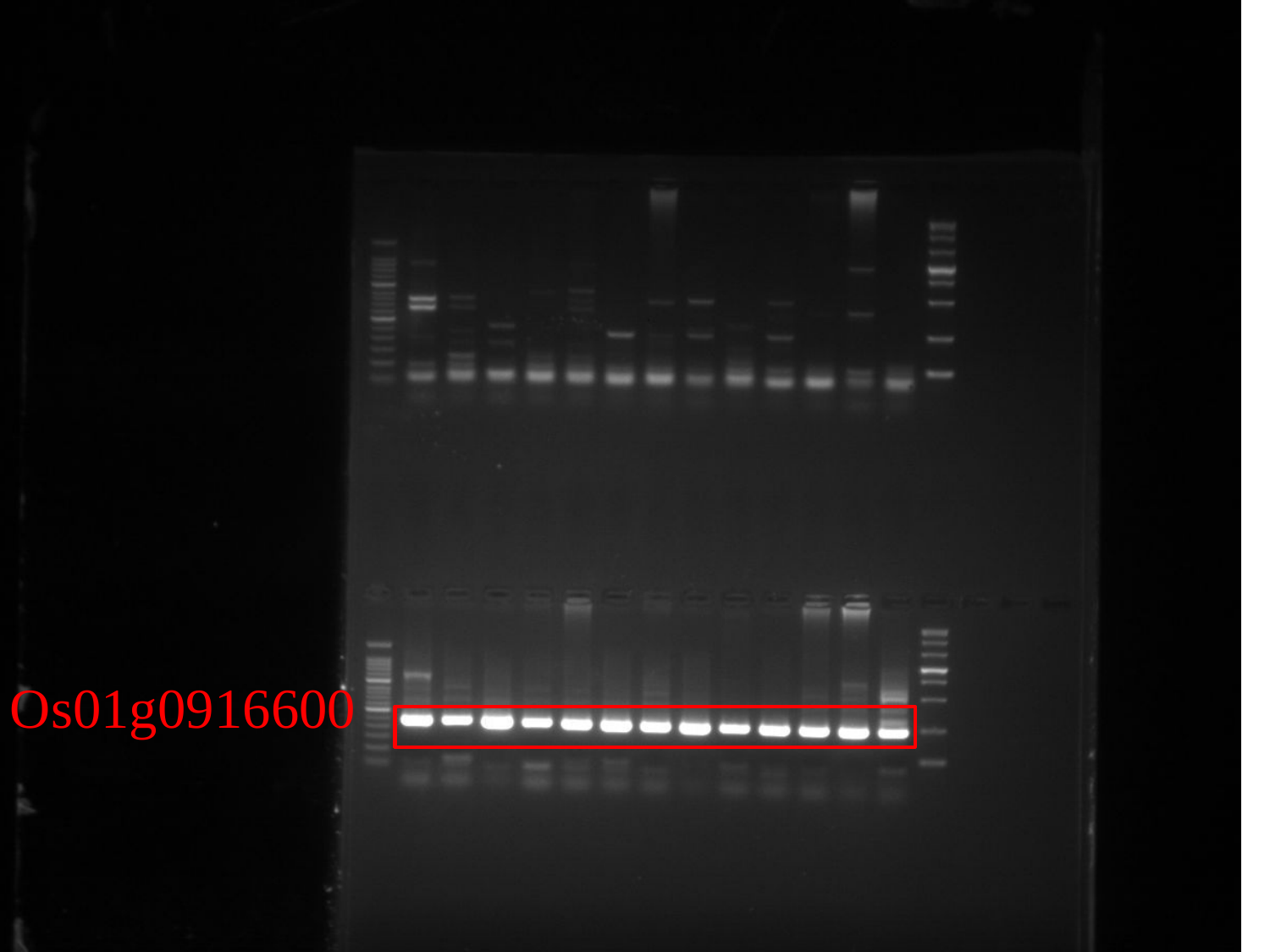

Os01g0916600

## Slide 21
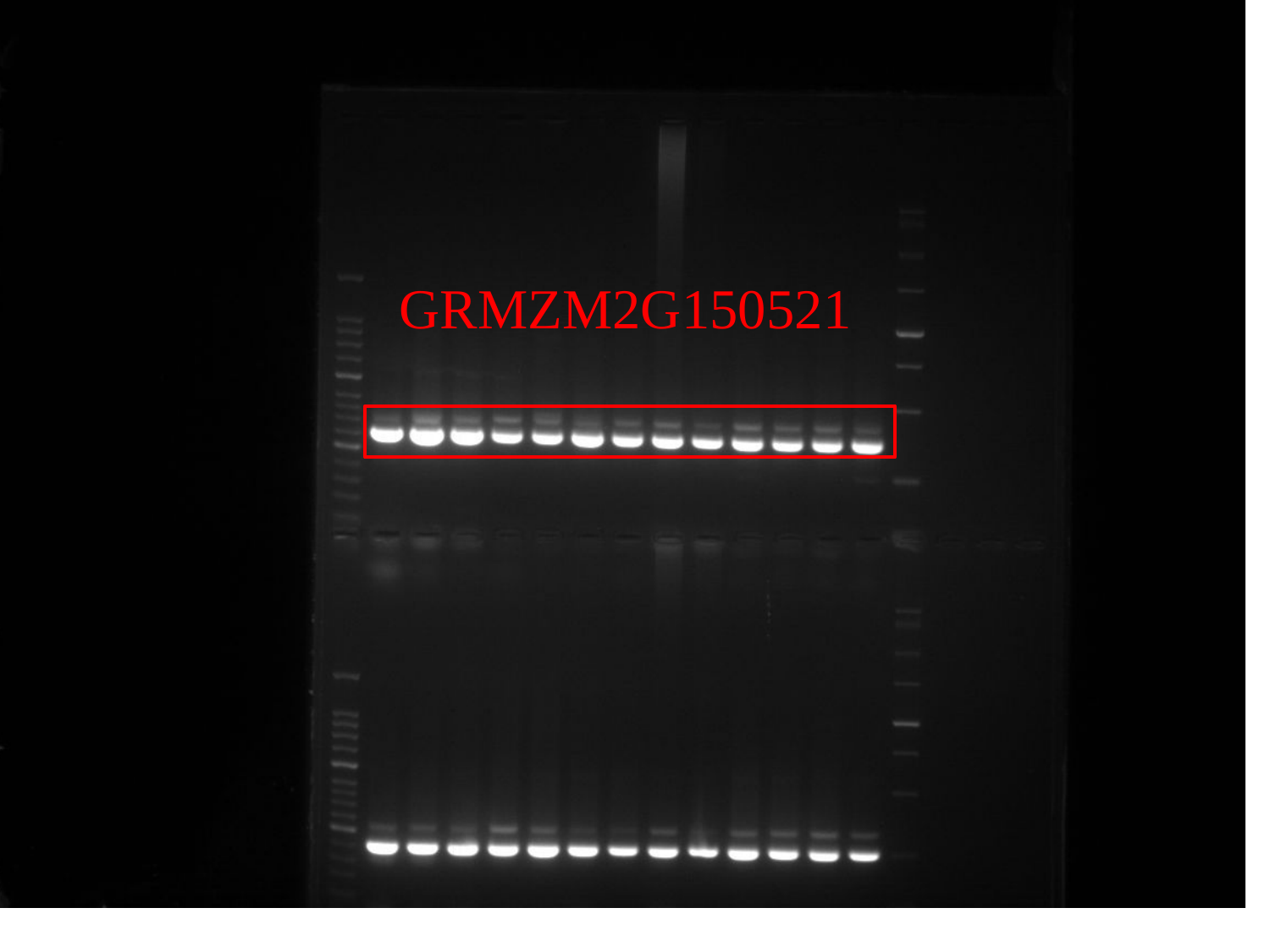

GRMZM2G150521

## Slide 22
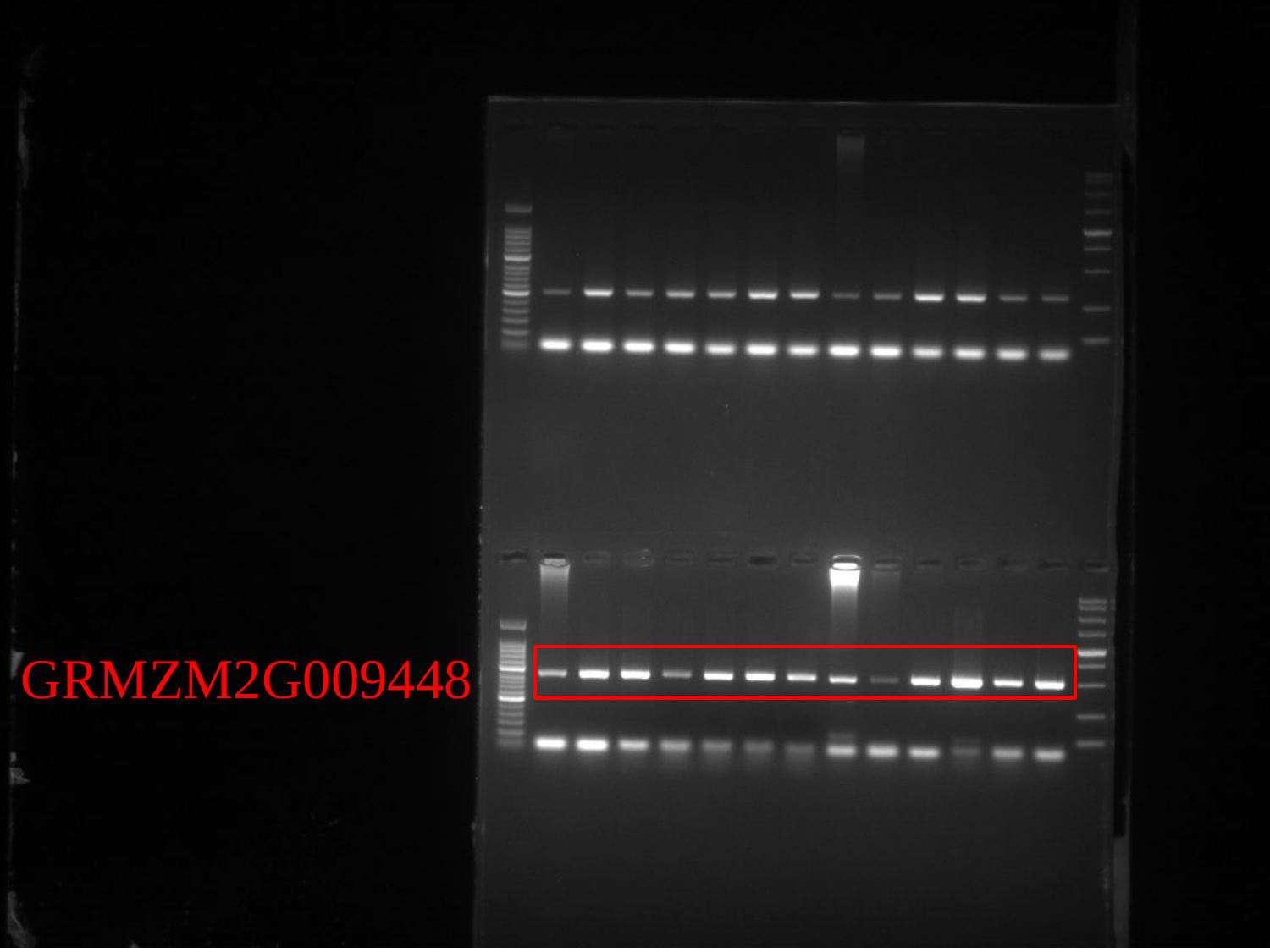

GRMZM2G009448

## Slide 23
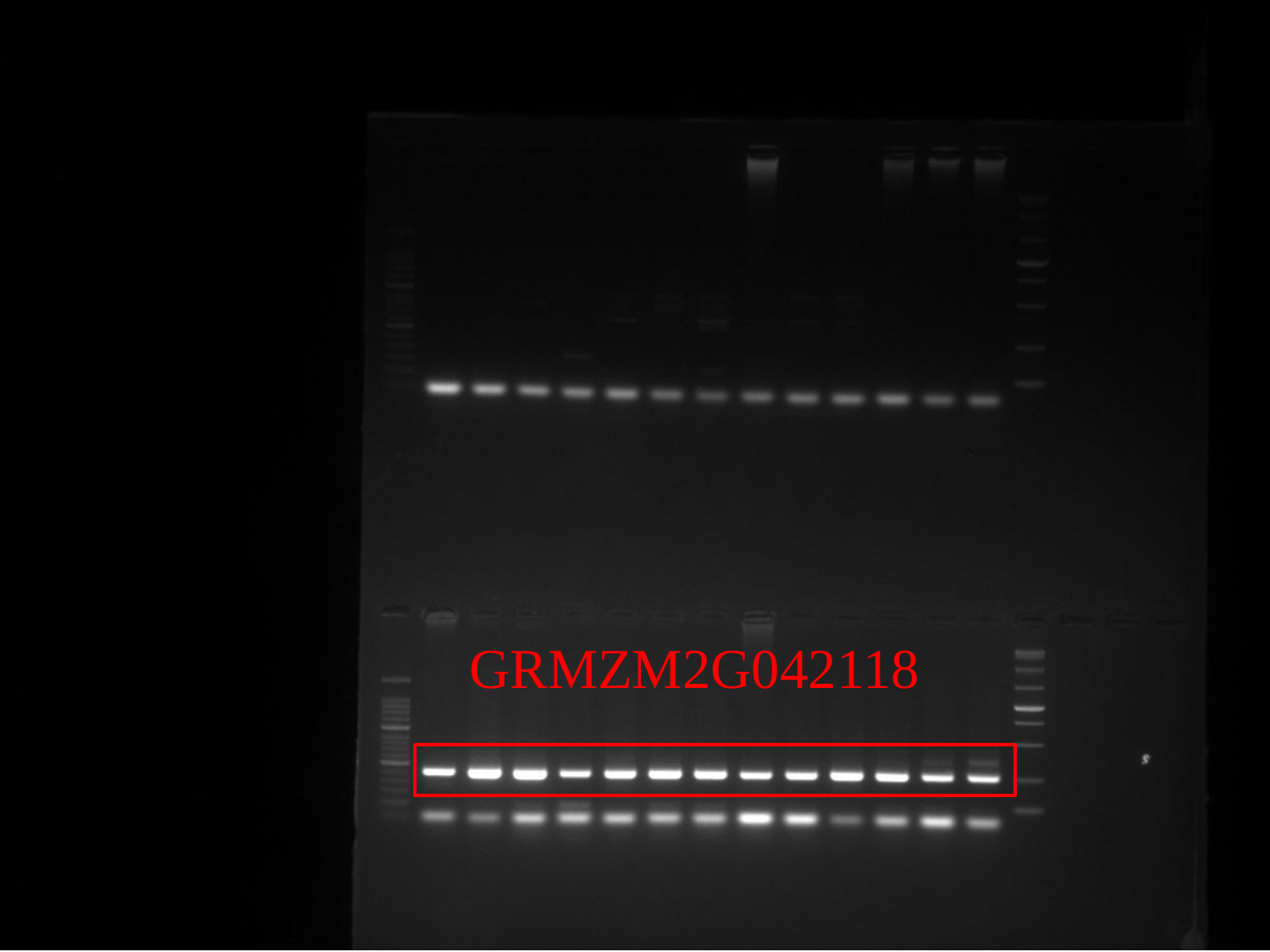

GRMZM2G042118

## Slide 24
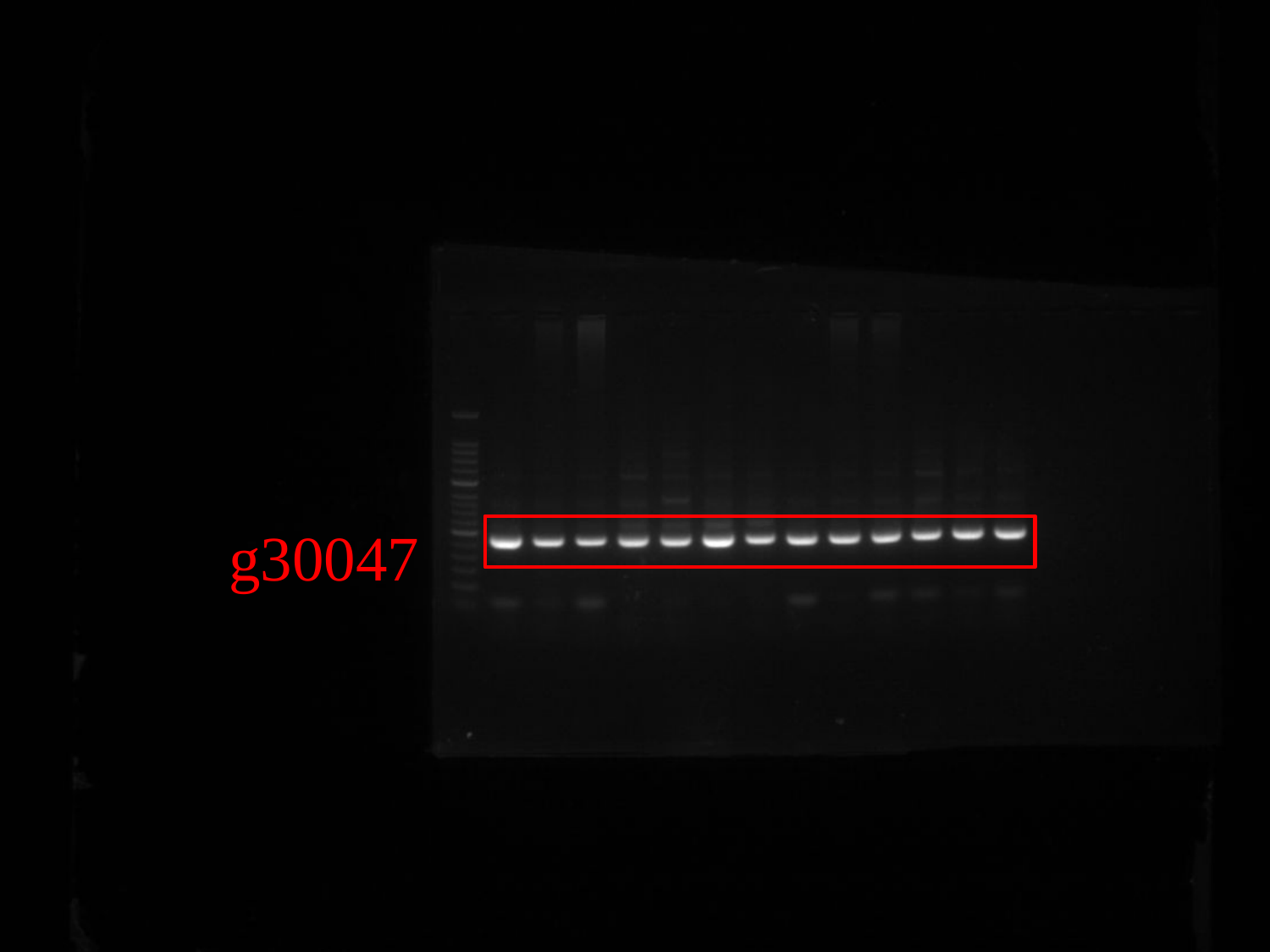

g30047

## Slide 25
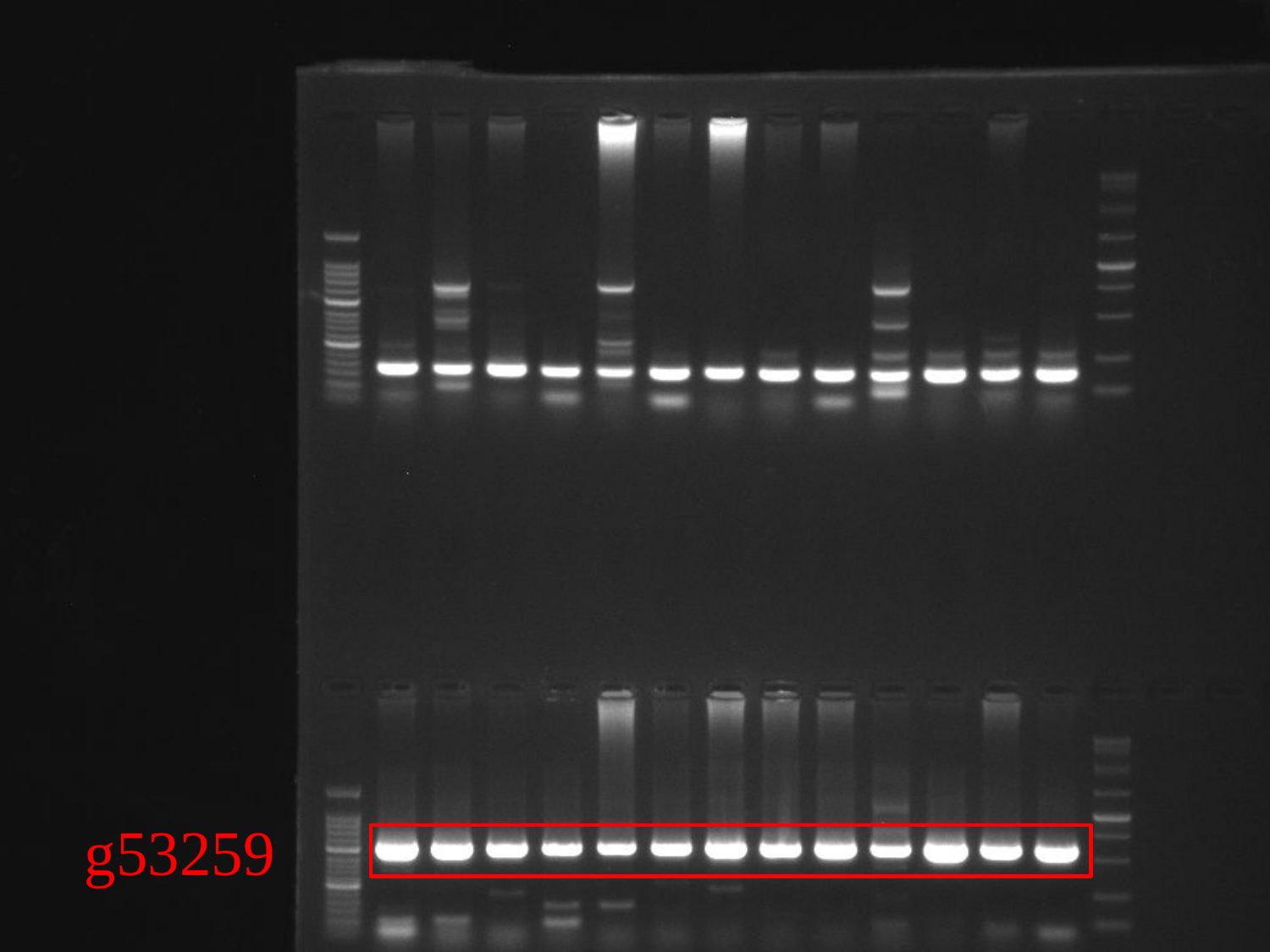

g53259

## Slide 26
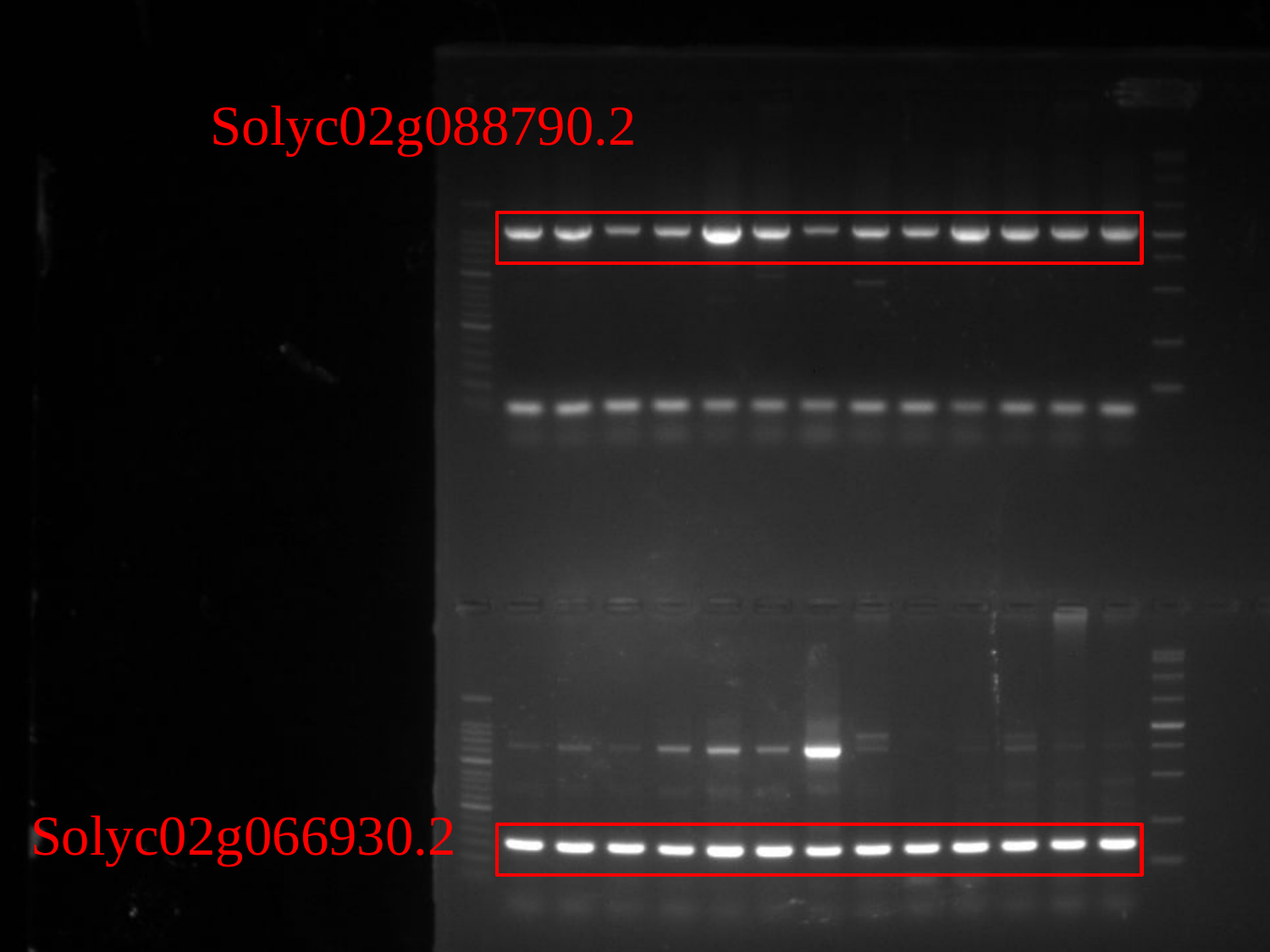

Solyc02g088790.2
Solyc02g066930.2

## Slide 27
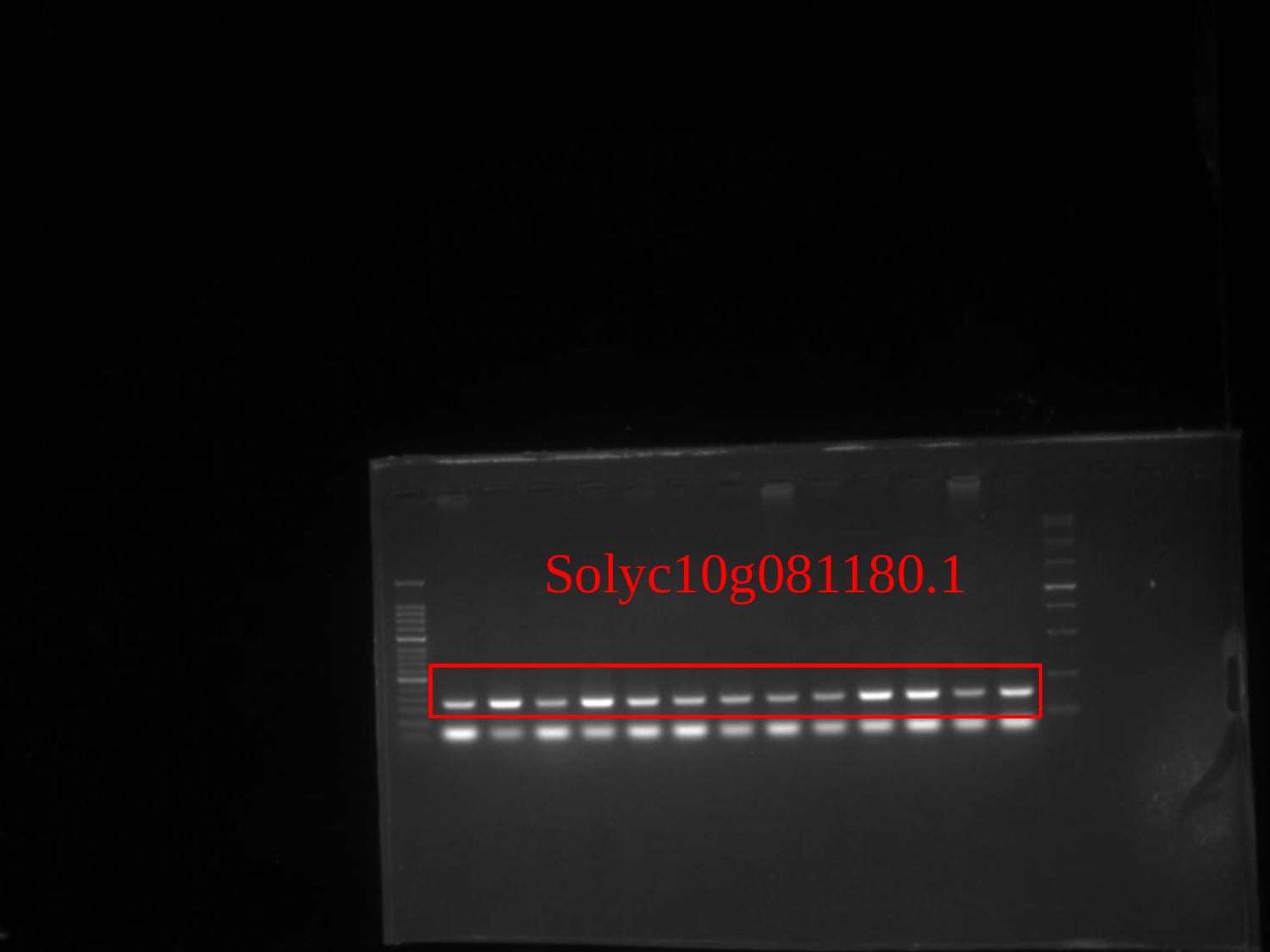

Solyc10g081180.1

## Slide 28
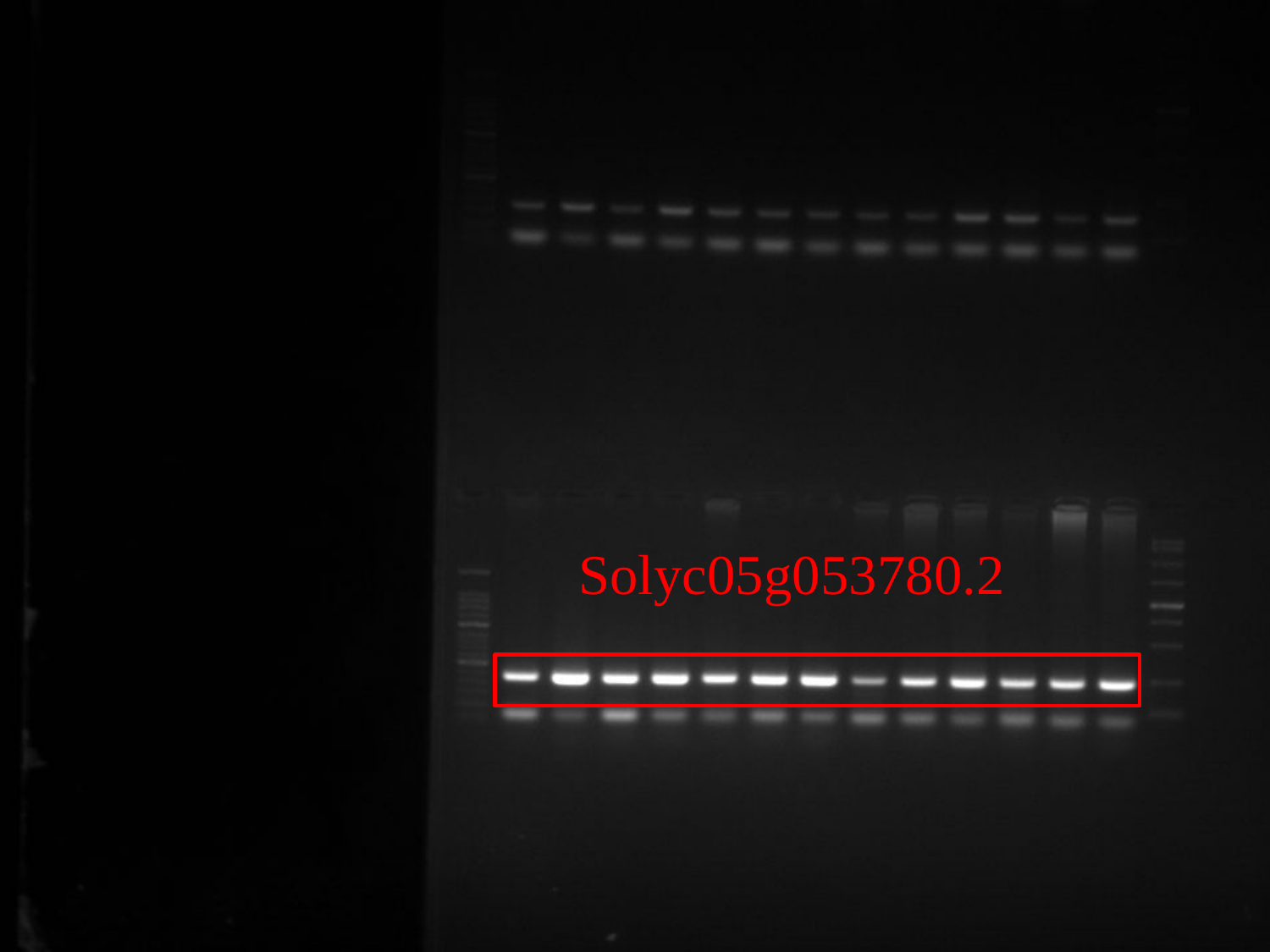

Solyc05g053780.2

## Slide 29
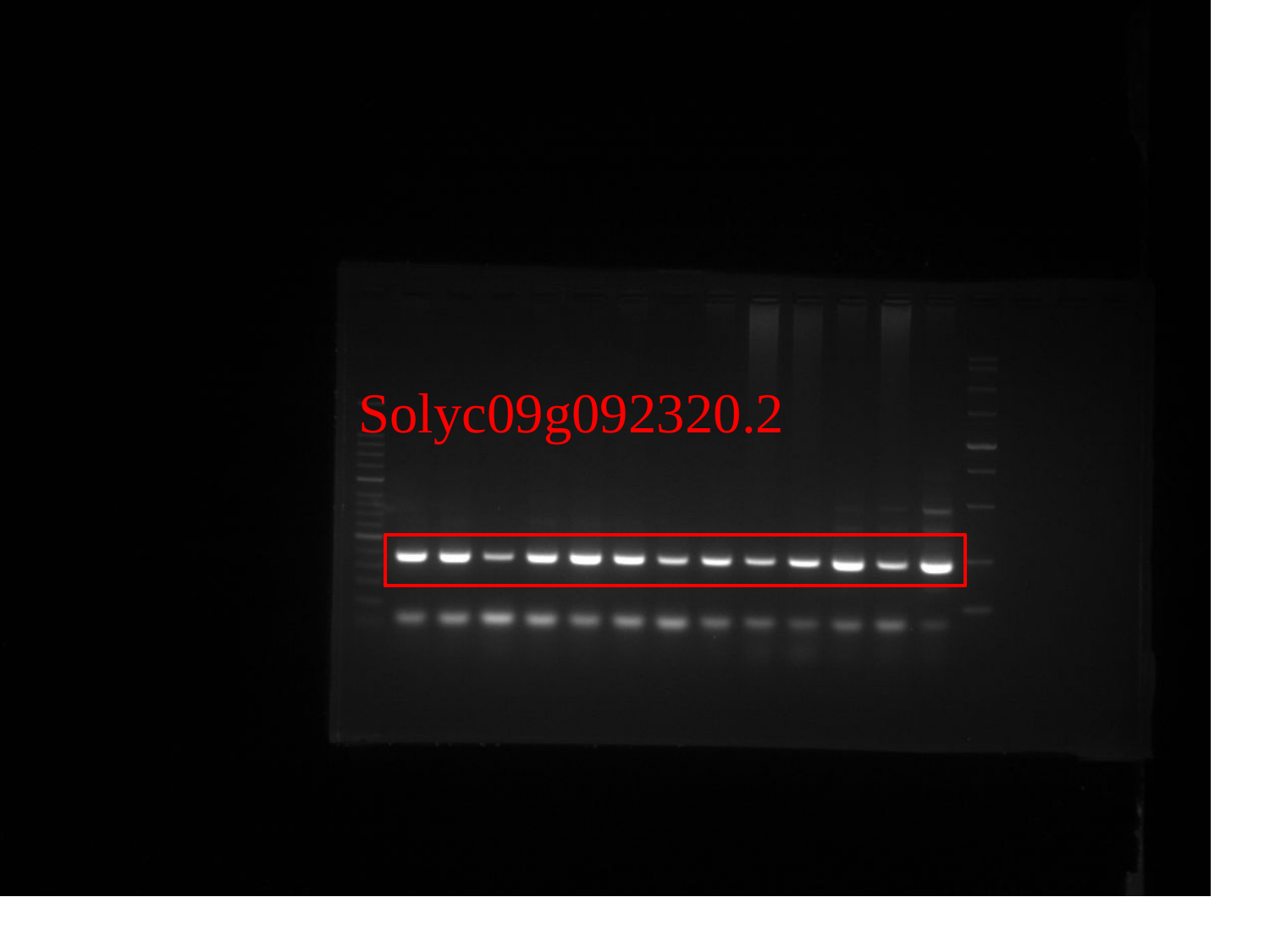

Solyc09g092320.2

## Slide 30
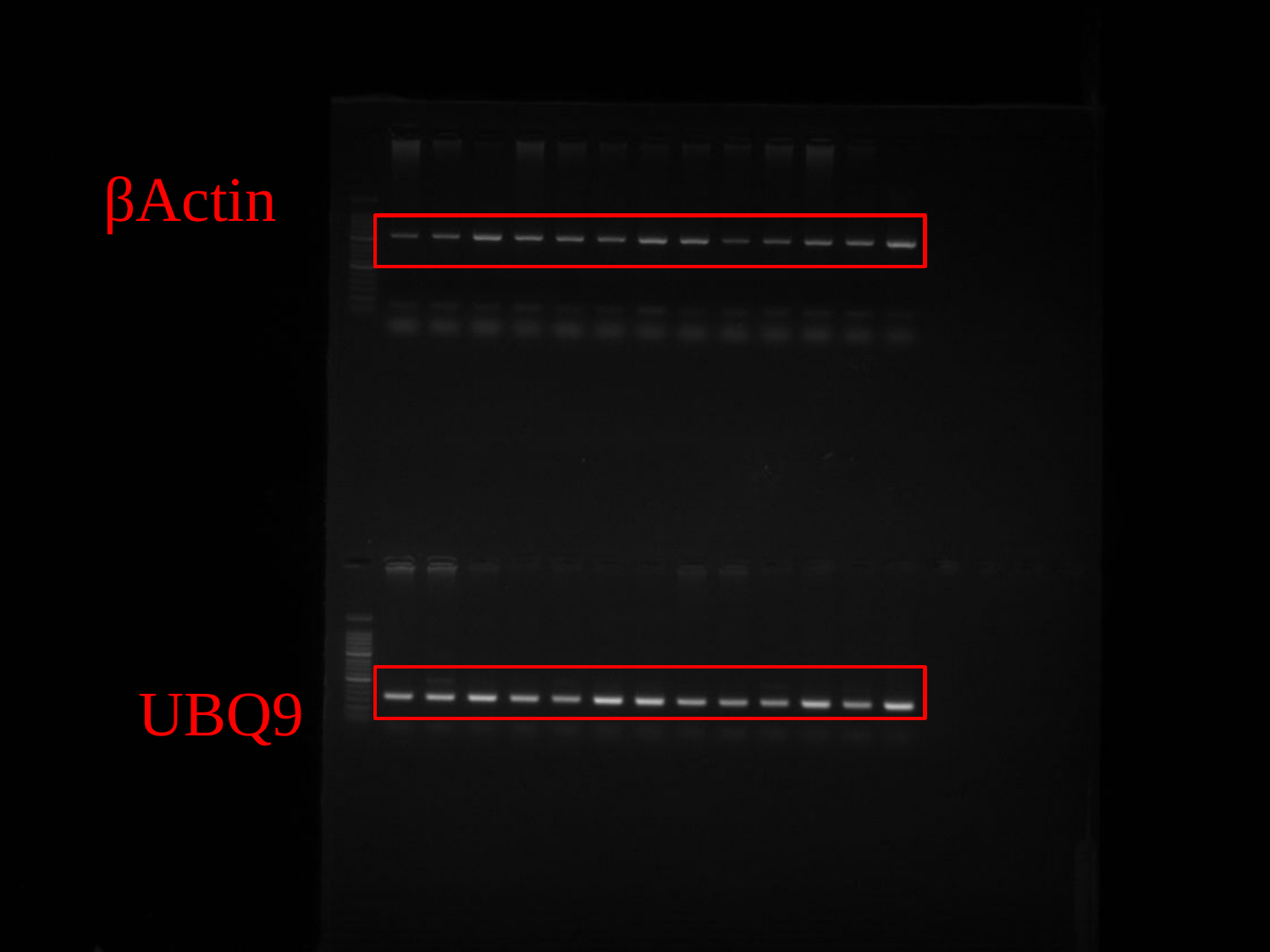

βActin
UBQ9

## Slide 31
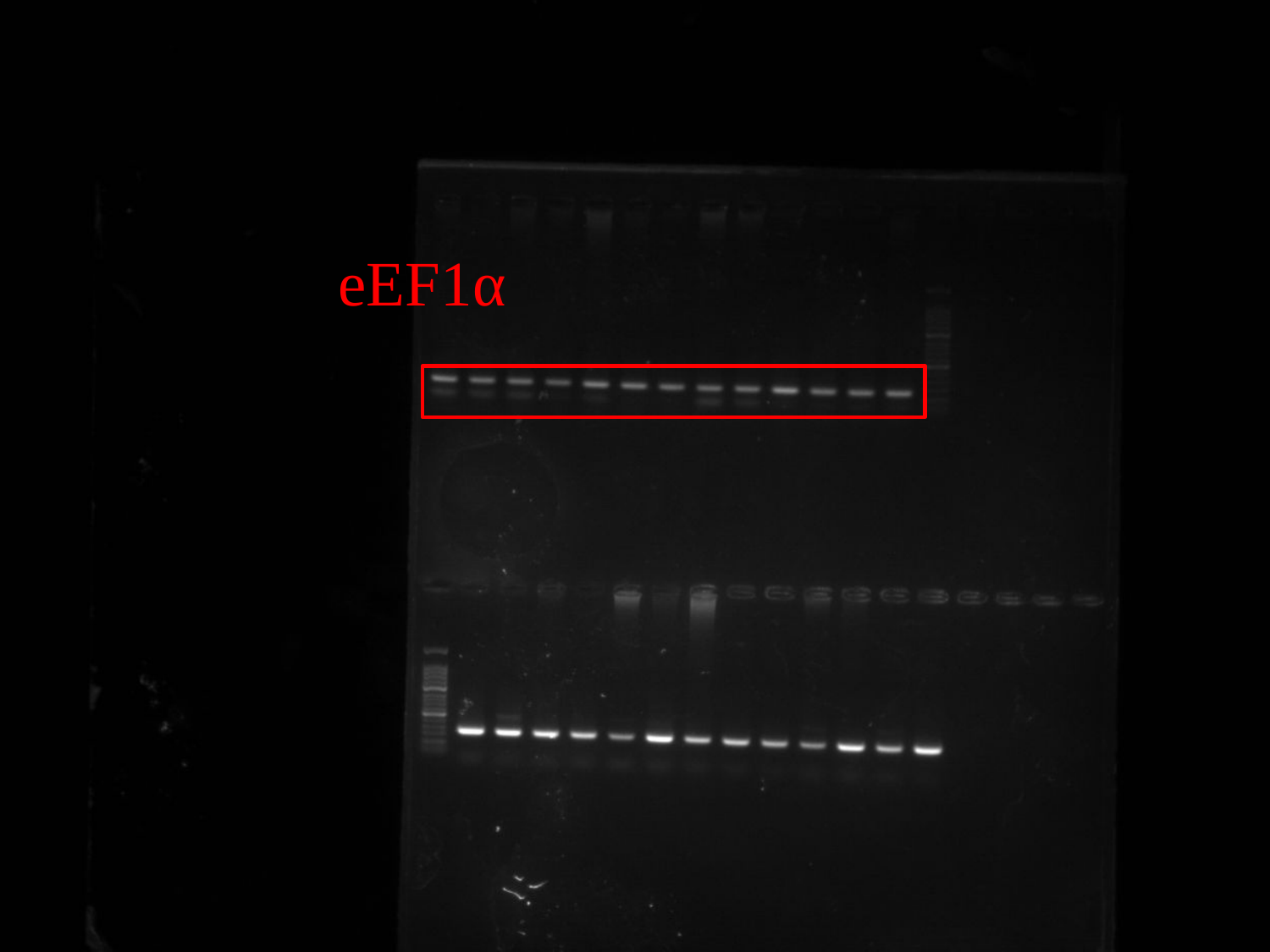

eEF1α

## Slide 32
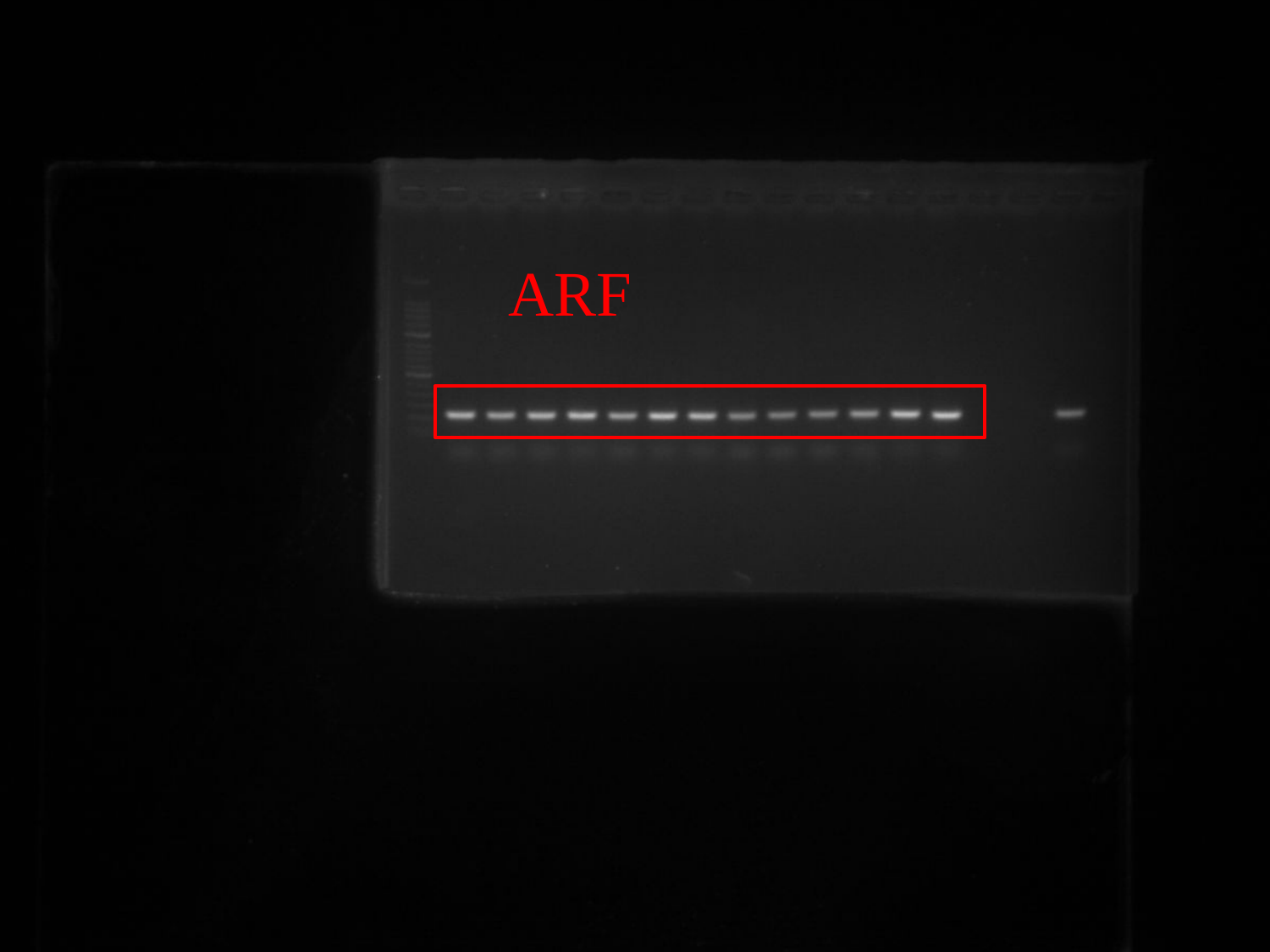

ARF

## Slide 33
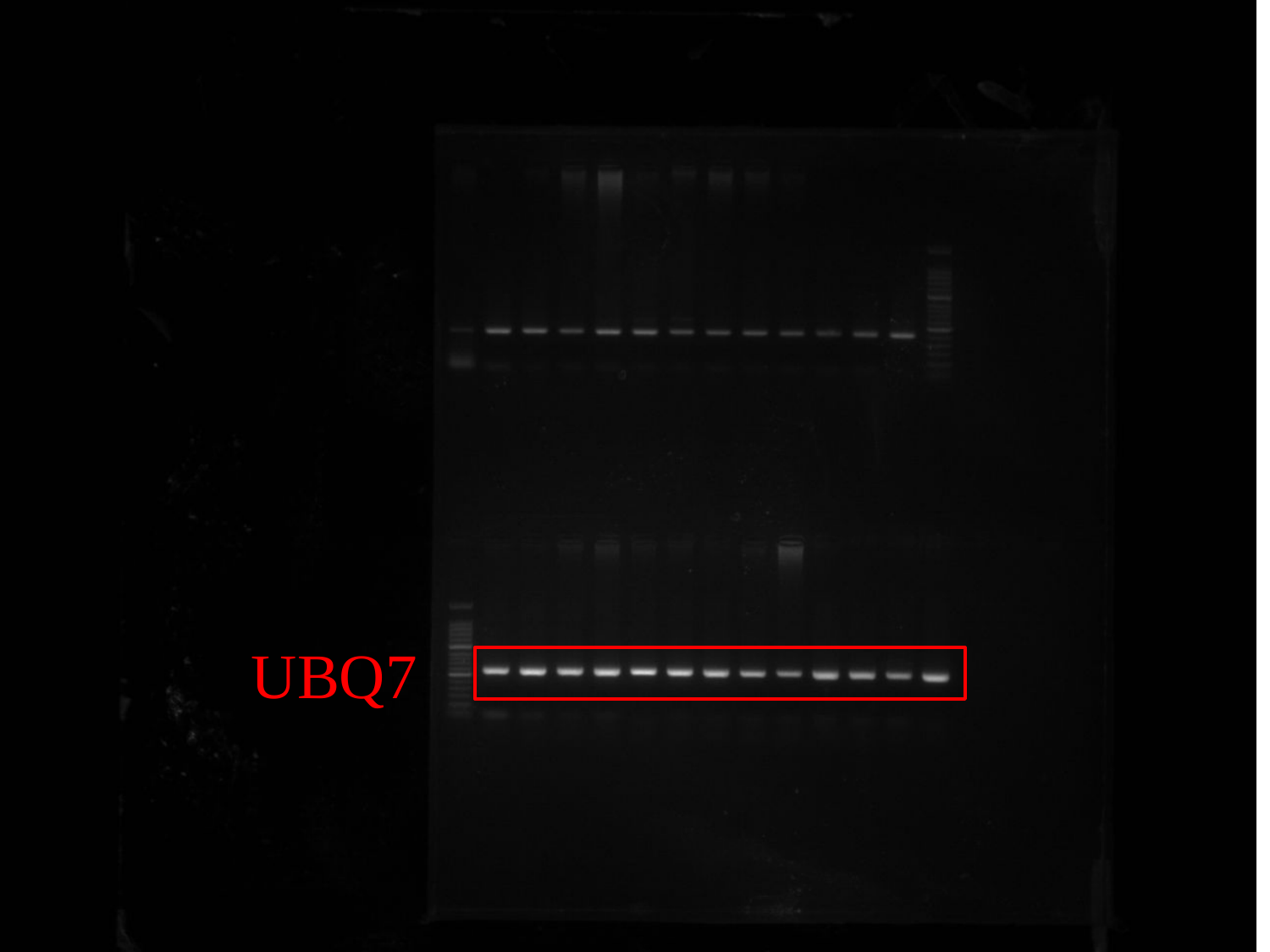

UBQ7
